# eNAMPT/Ac-STAT3/DIRAS2 Axis Promotes Development and Cancer Stemness in Triple-Negative Breast Cancer by Enhancing Cytokine Crosstalk Between Tumor-Associated Macrophages and Cancer Cells

**DOI:** 10.7150/ijbs.103723

**Published:** 2025-02-18

**Authors:** Lifen Zhang, Lu Wang, Ziyao Xu, Xingmei Zhang, Shaoyu Guan, Zhe Liu, Shanzhi Gu, Lin Zhao, Weichao Bai, Tian Li, Xinhan Zhao

**Affiliations:** 1 Department of Oncology, The First Affiliated Hospital of Xi'an Jiaotong University, Xi'an 710061, China.; 2 Senior Department of General Surgery, the First Medical Center of Chinese PLA General Hospital, Beijing 100853, China.; 3 College of Medical Technology, Chengdu University of Traditional Chinese Medicine, Chengdu 610000, China.; 4 Pharmaceutical Sciences Research Division, Department of Pharmacy, Medical Supplies Centre of PLA General Hospital, Beijing 100853, China.; 5 Department of Pancreatic-Biliary Surgery, The First Hospital of China Medical University, Shenyang 110001, China.; 6 Department of Forensic Medicine, Xi'an Jiaotong University, Xi'an 710061, China.; 7 Tianjin Key Laboratory of Acute Abdomen Disease-Associated Organ Injury and ITCWM Repair, Institute of Integrative Medicine of Acute Abdominal Diseases, Tianjin Nankai Hospital, Tianjin Medical University, Tianjin 300100, China.

**Keywords:** Cancer stemness, Extracellular nicotinamide phosphoribosyltransferase, Metastasis, Tumor-associated macrophages, Triple-negative breast cancer

## Abstract

The intricate relationship between tumor-associated macrophages (TAMs) and cancer cells is pivotal for carcinogenesis, with TAMs being integral to the tumor microenvironment (TME). This study explores the novel mechanisms by which TAMs regulate the progression of triple-negative breast cancer (TNBC) within the TME. Using a co-culture system and methodologies such as cytokine arrays, proteomics, and CRISPR-Cas9, we investigated the crosstalk between TAMs and TNBC cells. We found that high levels of CD163^+^ TAMs in TNBC tissues correlate with poor prognosis. TNBC cell-conditioned medium induces macrophage polarization towards the M2 phenotype, enhancing TNBC cell migration, invasion, and stemness through the secretion of extracellular nicotinamide phosphoribosyltransferase (eNAMPT). eNAMPT binding to CCR5 on TNBC cells activates STAT3, leading to the downregulation of the tumor suppressor DIRAS2 and an increase in CCL2, which promotes a macrophage recruitment loop. Intervention at the eNAMPT/CCR5 or CCL2 level disrupts this loop, mitigating TAM-induced effects. Our findings uncover a cytokine communication mechanism between immune and cancer cells, suggesting potential targets for TNBC detection and treatment.

## 1. Introduction

The prevalence of breast cancer has risen to become the leading malignant tumor among women globally, with the United States reporting the highest incidence, representing approximately 32% of all female cancers 1-5. Triple-negative breast cancer (TNBC), characterized by the absence of hormone receptors and human epidermal growth factor receptor 2 (HER2), is particularly aggressive. It is known for its resistance to standard endocrine and targeted therapies, as well as a propensity for local recurrence and distant metastasis 6. It is estimated that only 12% of patients with metastatic TNBC survive for five years after diagnosis, and even with treatment, the median overall survival is between 12 and 18 months 7. This underscores the urgent need for novel therapeutic strategies to improve outcomes for TNBC patients.

Epithelial-mesenchymal transition (EMT) is a pivotal cellular process driven by EMT-inducing transcription factors (EMT-TFs), which lead to the loss of epithelial characteristics and the acquisition of mesenchymal traits 8. There is a growing body of evidence indicating that EMT plays a significant role in tumor progression, facilitating infiltration and metastasis, and is implicated in the enhancement and preservation of cancer stem cell (CSC) characteristics 9-11. TNBC is characterized by a high enrichment of CSCs, which are instrumental in promoting chemoresistance, metastasis, and recurrence. The unique molecular mechanisms that facilitate the crosstalk between TNBC cells and the tumor microenvironment (TME) are crucial for maintaining the CSC-like properties 12. Understanding the intricate mechanisms that underlie EMT, the stemness of TNBC, and its aggressive behavior is essential.

The TME is a complex milieu rich in cells and cytokines that are vital for tumor growth, invasion, and metastasis 13. Within the TME, macrophages are the most abundant immune-related cells, exhibiting diverse phenotypes and functionalities influenced by stimuli from cancer and stroma cells 14. They can polarize into M1 or M2 macrophages, with tumor-associated macrophages (TAMs) typically aligning with the M2 phenotype 15, 16. Monocytes and macrophages are actively recruited to the tumor site and differentiate into TAMs upon induction by factors such as IL-6, CCL2, and GM-CSF present in the TME. They interact with tumor cells by releasing cytokines, thereby influencing tumor progression 17. TAMs can further modulate immune evasion or stimulate antitumor immunity through the secretion of soluble factors and expression of cell surface proteins 18. Specific examples of TAM activity include the production of CCL22, which has been shown to induce focal adhesion kinase, promote metastasis, and decrease survival rates in esophageal squamous cell carcinoma 19. In gastric cancer, TAMs have been observed to highly express PD-L1 and secrete CXCL8, inhibiting CD8^+^ T cell function and infiltration 20. Despite these insights, the precise mechanisms by which TAMs regulate EMT and the malignant behavior of TNBC remain to be fully elucidated, highlighting the need for further research in this area.

Despite recent advances in understanding the molecular underpinnings of TNBC, current treatment options remain limited, primarily due to the aggressive nature of the disease and its lack of targeted therapeutic vulnerabilities, emphasizing an urgent need for novel therapeutic strategies to improve patient outcomes. The complex and bidirectional relationship between TAMs and TNBC cells is a pivotal factor in the TME, exerting profound influences on cancer progression and patient prognosis. This study aims to dissect the underlying mechanisms by which TAMs contribute to the aggressive phenotype of TNBC. Utilizing a combination of co-culture systems, cytokine array analysis, and molecular biology techniques such as CRISPR-Cas9 gene editing, we will elucidate the signaling pathways activated by eNAMPT, including the phosphorylation and acetylation of STAT3. We will assess the impact of these pathways on EMT, CSC properties, and immune cell recruitment. By targeting the TME and disrupting the pro-tumorigenic interactions that fuel TNBC progression, our research has the potential to significantly improve treatment strategies and outcomes for patients afflicted with this aggressive form of breast cancer.

## Materials and Methods

### Cell culture and reagents

The human TNBC cell lines (MDA-MB-231 and SUM159), human monocyte cell line THP-1, and normal breast epithelial cell line (MCF-10A) were acquired from Procell Life Science & Technology (Wuhan, China). These cell lines were cultured in the following media: Leibovitz's L15 medium (KeyGen, Nanjing, China), DMEM/F12 (HyClone, Logan, UT, USA), and RPMI-1640 (KeyGen, Nanjing, China) supplemented with 10% fetal bovine serum (FBS) and 1% penicillin/streptomycin. All cells were cultured in an incubator at 37°C and 5% CO_2_. Phorbol 12-myristate 13-acetate (PMA) (#MB5349, Meilunbio, Dalian, China) was solubilized in DMSO and stored at -20°C. Recombinant human NAMPT was purchased from Enzo Life Sciences. IL-4 (#214-14) and IL-13 (#210-13) were procured from PeproTech (USA), reconstituted in water and diluted in phosphate-buffered saline (PBS) containing 5% trehalose. Anti-CCL2 neutralizing antibody (#MAB679) was sourced from R&D Systems (Wiesbaden, Germany). The STAT3 antagonist Stattic (#T6308) was purchased from Topscience (Shanghai, China).

According to the previous literature 21, THP-1 cells exposed to PMA (100 ng/mL) for 24 hours produced M0 macrophages. THP-1 cells were stimulated with PMA (100 ng/mL) for 6 hours, and then their differentiation was aided for 18 hours by IL-4 (20 ng/mL) and IL-13 (20 ng/mL). This resulted in the production of M2 macrophages. Subsequently, after successfully differentiating, the macrophages were cultivated in fresh medium for 48 hours and the supernatant was collected, filter, and obtained the corresponding conditioned medium (M0-CM and M2-CM). A 6-well plate was utilized to hold a 0.4 μm Transwell chamber (Millipore, Billerica, USA) for co-culturing macrophages and TNBC cell lines. Following a 48-hour co-cultivation period, TAMs or TNBC cells were harvested for additional examination. eNAMPT (20 nM) or the STAT3 inhibitor Stattic (10 μM), was added to the lower compartment or pre-starved TNBC cells. Based on drug-action half-life, the experiment lasted between 6 and 24 hours.

### Construction of lentivirus or plasmids

For TNBC (MDA-MB-231 and SUM159) cells, LV-GFP-shNC and LV-GFP-shCCR5 lentiviruses from Hanbio (Shanghai, China) were used at an MOI of 10. For THP-1 cells, LV-GFP-shNC and LV-GFP-shNAMPT lentiviruses from Hanbio were used at an MOI of 30. Plasmids hSTAT3-pcDNA3.1-HA, hSTAT3-pcDNA3.1-HA K685R, and hSTAT3-pcDNA3.1-Flag and empty vector were constructed by Biokeeper (Xi'an, China) and validated by sequencing. LentiCRISPR V2_U6-DIRAS2 sgRNA (human)-EFS-hCas9-2A-Puro and control group were purchased from Beijing Syngentech. The gDNA sequences were as follows: gDNA1, GCAGAGTAAGGATTACCGGGTGG; gDNA2, GGAGAGCTACATCCCGACGGTGG; gDNA3, CCCATCTACGAACAAATCTGCGA. Transfections were conducted using Lipo8000^TM^ reagent (Beyotime). After incubation for 72 hours, cells were treated with puromycin or direct follow-up testing.

### Western blot and Co-immunoprecipitation (Co-IP)

For protein extraction, cells were broken down using RIPA lysis buffer with protease and phosphatase inhibitors (Solarbio, Beijing, China). The lysate mixtures were then centrifuged at 12,000g for 15 minutes at 4°C. Protein concentration in the resulting lysates was analyzed using a BCA Protein Assay Kit (Beyotime, Shanghai, China). For western blot, denatured proteins (20-30 μg per lane) were separated via a 10% sodium dodecyl sulfate-polyacrylamide gel electrophoresis (SDS-PAGE), before transferring to a PVDF membrane (Millipore, Boston, MA, USA). The membrane was exposed to block with 5% skim milk for 1 hour. The membrane was then incubated overnight with properly diluted primary antibodies. The next step involved a 1-hour incubation with horseradish peroxidase (HRP)-conjugated secondary antibodies (Immunoway, California, USA). The final step of the process was the visualization of the protein bands using an enhanced chemiluminescence (ECL) system.

For Co-IP, cells were performed using the same lysis procedure as described above. After determining the protein concentration, equal amounts of protein samples were mixed with protein A/G agarose beads (Santa Cruz Biotechnology, USA) and either primary antibody or a control immunoglobulin (IgG) overnight. Western blot was used to examine the precipitates after they had been thoroughly washed.

The following antibodies were used in the western blot process: antibodies against JAK2 (#3230), Phospho-JAK2 (Tyr1007/1008) (#3771), Phospho-STAT3 (Tyr705) (#9145), E-cadherin (#3195), and N-cadherin (#14215) from Cell Signaling Technology (Boston, MA, USA), Anti-CCR5 (#ab7346) from Abcam (Cambridge, UK), Anti-GAPDH (#sc-47724) from Santa Cruz Biotechnology (USA), Acetyl-STAT3 (Lys685) (#YK0175) from Immunoway (Suzhou, China), Anti-Arginase1 (#66129-1-Ig), anti-DIRAS2 (#15557-1-AP), anti-STAT3 (#10253-2-AP), anti-Flag (#66008-4-Ig), anti-OCT4 (#60242-1-Ig), anti-SOX2 (#66411-1-Ig), anti-Nanog (#67255-1-Ig), anti-HA (#66006-2-Ig), and anti-Snail (#13099-1-AP) from Proteintech (Wuhan, China).

### Quantitative reverse transcription PCR (RT-qPCR)

To extract total RNA, the RNAfast 200 kit (Fastagen, Shanghai, China) was used, following its instructions. Then, using the PrimeScript RT reagent kit (TaKaRa, Dalian, China), RNA samples were reverse transcribed into cDNA. The SYBR Green PCR Premix Ex TaqTM kit (TaKaRa) was utilized to perform RT-qPCR in order to quantify the target gene. Table 1 lists the specific primers that were utilized for RT-qPCR.

### Wound healing and Transwell invasion assay

Cells were seeded and cultured to full confluence in 6-well plates for the wound-healing experiment. A 200 μL pipette tip was used to scrape the cell monolayer after it had been starved for 24 hours in a serum-free media. Using a microscope, pictures were collected at 0, 24, and 48 hours to track cell migration to the injured area. Utilizing Transwell chambers (8 μm, Millipore) placed within a 24-well plate, the Transwell invasion assay was performed. Serum-free media or macrophage-derived CM was introduced to the lower chamber after cells were plated in the upper chamber. 4% formaldehyde fixation and crystal violet staining were applied to the invading cells following a 24-48-hour incubation period. The image and number of invasive cells were then recorded under five visual fields.

### Microsphere formation

After seeding TNBC cells at a density of 1,000 cells per well on 6-well ultralow attachment plates (Corning, NY, USA), the cells were grown for 10-14 days in serum-free DMEM/F12 mix supplemented with 20 ng/mL human FGF, 10 ng/mL human EGF and 1% B27. Fresh medium and conditioned medium or eNAMPT were added every two days. Finally, we counted the number of tumor microspheres and measured their sizes.

### Flow cytometry

For detecting CD163 proteins, cells were collected and washed. Subsequently, they were resuspended in 100 μL of PBS solution with 2% FBS in it. Next, the PE-conjugated rabbit anti-human CD163 antibody (BD, NY, USA) and corresponding isotype control were added to the cell suspension. For 40 minutes, cells were kept cold (on ice) and in the dark. After incubation, cells were washed, and flow cytometry analysis was conducted on a BD FACScelesta instrument to detect the expression of CD163. Thereafter, FlowJo software was utilized for the data analysis.

### Cytokine analysis

A human cytokine array kit containing 36 distinct cytokine antibodies was purchased from R&D Systems. In short, supernatants from cell cultures were gathered. Specialized buffers blocked the antibody array membranes. Next, antibody arrays were treated with cell supernatants at 4°C for an entire night. The membranes were incubated with streptavidin-HRP for 30 minutes at room temperature following three washing. Ultimately, the signals were identified using the ECL advance reagent, and ImageJ was used to quantify them.

### Dual-luciferase reporter assay

After seeding MDA-MB-231 cells (1×10^5^) in 24-well plates for a whole night, the cells were co-transfected using Lipo8000^TM^ with the specified plasmid or corresponding negative control and wild-type or mutant luciferase reporter vectors. Following a 48-hour transfection, luciferase activities were assessed in accordance with the manufacturer's protocol using the Dual-Luciferase Reporter Gene Assay Kit (Beyotime).

### Chromatin-immunoprecipitation (ChIP)

Chromatin samples from the MDA-MB-231 cells, including those with STAT3 mutations, were broken down to have an average DNA length between 200-500bp. The samples were incubated with a primary antibody against STAT3 overnight at 4°C. This was followed by the addition of Protein A/G Agarose beads to isolate the immune-complexes. After washing and removing the beads, the crosslink between the DNA and proteins were reversed by heating up to 65°C for 4 hours. The DNA was then eluted, and detection of the DIRAS2 DNA in the immunoprecipitated chromatin and input was performed via RT-qPCR.

### DNA methylation analysis of the DIRAS2

The DIRAS2 promoter's methylation state was assessed in each cell line using pyrosequencing analysis. Genomic DNA was extracted from the cell lines using the QIAamp DNA Mini Kit and treated with bisulfite. This treatment changed unmethylated cytosines to uracils, while methylated cytosines remained unchanged. After digestion, the residual DNA was measured using RT-qPCR. The following primers were used for pyrosequencing: CpG-F, GGTAATTTTTTTTTAGGGGTTGGTAAATA and CpG-R, AAAAAAACCCCATCCACACACCCT.

### Animal experiments

All experiments involving animals were conducted according to the ethical policies and procedures approved by the Animal Care and Use Committee of Xi'an Jiaotong University. The study was reported according to the ARRIVE guidelines. Four-week-old female BALB/c mice were fed in a facility that adheres to specific pathogen-free (SPF) standards. Approximately 5 × 10^6^ MDA-MB-231 cells stably transfected with either non-targeting shRNA (shNC) or CCR5-targeting shRNA (shCCR5) were fully cultured to reach confluence. The cells were then trypsinized, resuspended, and aliquoted such that each 100 μL of cell suspension contained the desired number of cells. Mice were randomly selected and injected into their mammary fat pads (*n* = 5 per group). Every 3 days, the tumor volume was measured. When the tumors reached the desired size, all of the mice were sacrificed simultaneously with excess CO_2_, and the tumors were taken out for further analysis.

### 2.13 Immunohistochemistry (IHC)

The human TNBC tissue microarrays were prepared by Zhuolibiotech (Shanghai, China). Each paraffin block was sectioned, deparaffinized, and rehydrated. Primary antibodies were incubated on the sections for an entire night at 4°C. The FITC-labeled goat anti-rabbit IgG (H+L) antibody or HRP-conjugated secondary antibody (Sxyksw, Xi'an, China) was chosen as the secondary antibody, depending on the type of primary antibody. Following that, samples were stained with a DAB kit for IHC. The stained sections were scanned and independently evaluated by two pathologists blinded to samples.

### Statistical analysis

Data are presented as means ± standard error of mean (SEM) at least three independent experiments. The relationship between CD163 expression levels and clinical variables, as well as gene expression, was assessed using Pearson correlation analysis. For the comparison of continuous variables, one-way analysis of variance (ANOVA) or Student's *t*-test was applied, based on the data distribution. Survival prognosis was evaluated using the Kaplan-Meier method, and survival curves were constructed. The differences in survival between subgroups were statistically evaluated using the log-rank test. A two-tailed *P*-value < 0.05 was considered to indicate statistical significance.

## Results

### High CD163^+^ TAMs infiltration in TNBC tissues correlated with poor prognosis

**Figure 1:**
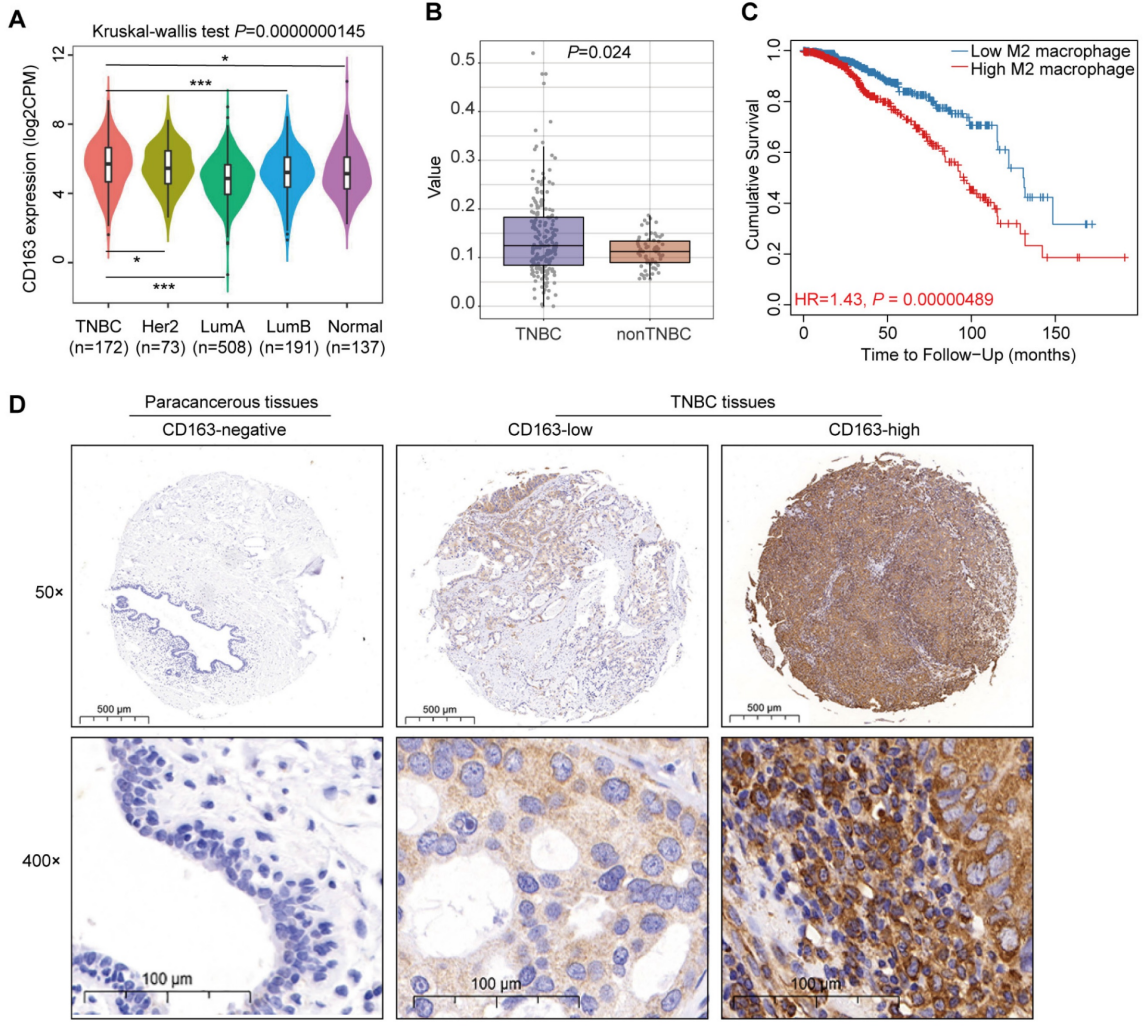
** Correlation between high M2 macrophage infiltration and poor prognosis in TNBC.** (A) Violin plots depicting CD163 expression in four distinct molecular subtypes of breast cancer and in normal tissue. (B) Box plots illustrating the infiltration value of M2 macrophages in TNBC and non-TNBC. (C) Correlation between M2 macrophage infiltration and overall survival in TNBC patients. (D) Representative IHC staining of CD163 in tissue sections from human TNBC and paracancerous samples. Tumor and paracancerous tissues are matched in 48 cases each. *P < 0.05, ***P < 0.001.

To elucidate the role of TAMs in TNBC progression and patient prognosis, we analyzed data from TCGA database, revealing that TNBC exhibit elevated CD163 mRNA expression relative to luminal A (*P* = 0.001), luminal B (*P* = 0.001), HER2-enriched (*P* = 0.043), and normal tissue (*P* = 0.017) (Fig. 1A). This finding underscores a potential link between TAMs and TNBC. Further analysis indicated that TNBC exhibit a higher infiltration of M2 macrophages compared to non-TNBC cases (Fig. 1B). Utilizing the TIMER public database, we observed a negative correlation between increased M2 macrophage infiltration and overall survival rates in TNBC patients (Fig. 1C). To validate these findings, we conducted IHC assessment of CD163 expression in a tissue microarray comprising 96 samples of TNBC and adjacent paracancerous tissues. Our results demonstrated significantly higher CD163 expression in TNBC tissues compared to paracancerous tissues (Fig. 1D). High CD163 expression was observed in 62.5% (30/48) of the TNBC samples, and this was significantly associated with age (*P* = 0.039) and TNM (*P* = 0.002), while no association was found with other clinicopathological parameters, such as Ki-67 expression (Table 2). Collectively, these data indicate that CD163^+^ TAMs are abundant in TNBC and may significantly contribute to tumor aggressiveness and adverse prognosis.

### TAM-mediated EMT promoted invasion, metastasis, and stemness of TNBC cells

**Figure 2:**
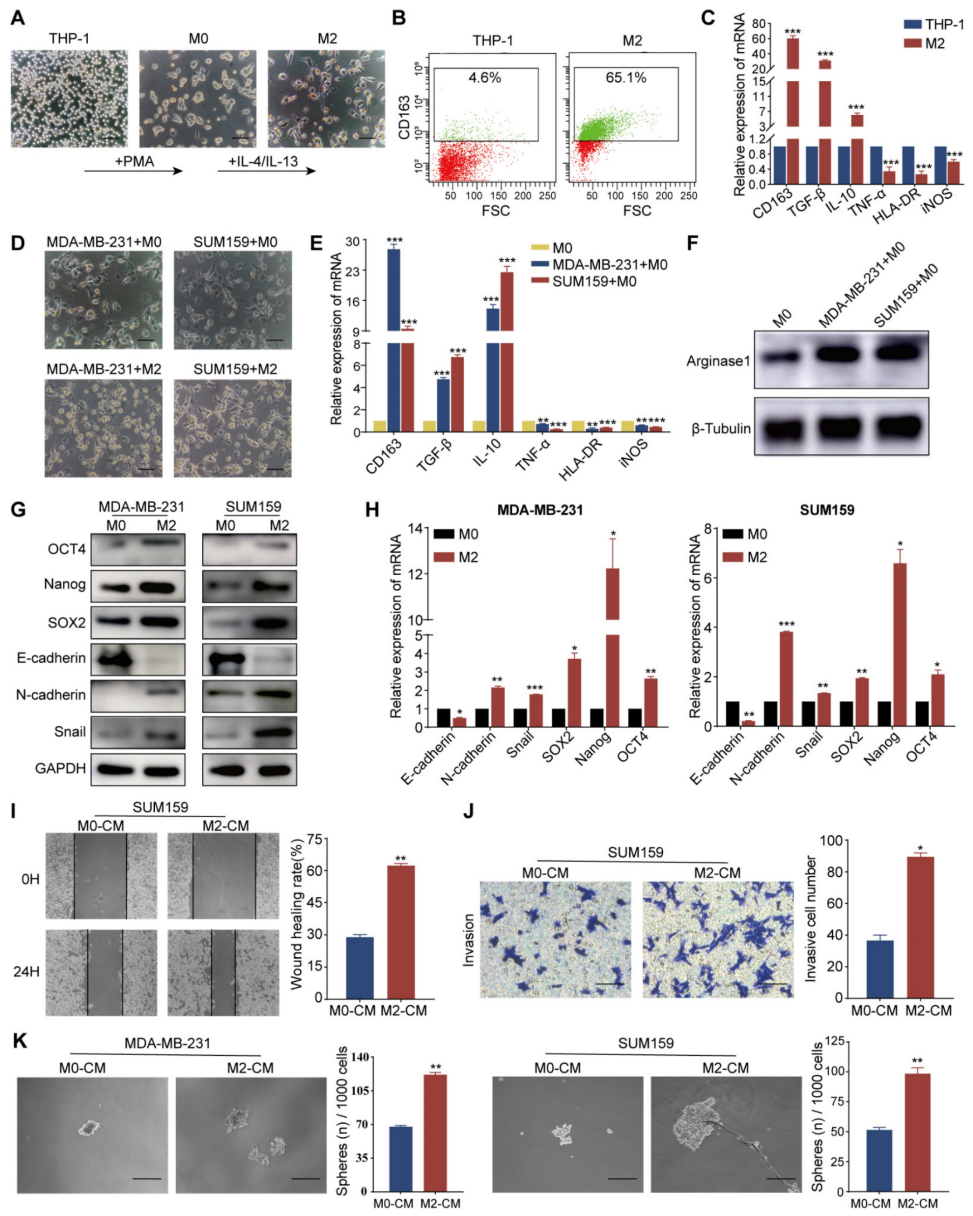
** TAMs induce EMT and enhance invasion, metastasis, and CSC-like properties of TNBC cells.** (A) Micrographs illustrating the morphological changes in macrophages induced by THP-1 cells (magnification, 100×, scale bar=100 μm). (B) Flow cytometry analysis of CD163 protein levels in THP-1 and M2 macrophages. (C) RT-qPCR analysis of mRNA levels of M2 markers (CD163, TGF-β, IL-10) and M1 markers (TNF-α, HLA-DR, iNOS) in THP-1 and M2 macrophages. (D) Morphology of M0 macrophages induced by THP-1 and co-cultured with MDA-MB-231 and SUM159 cells (magnification, 100×, scale bar=100 μm). (E) RT-qPCR analysis of macrophage markers expression in M0 macrophages cultured alone or co-cultured with MDA-MB-231 and SUM159 cells. (F) Western blot analysis of Arginase1 expression in M0 macrophages cultured alone or co-cultured with MDA-MB-231 and SUM159 cells. (G) The impact of TAMs on EMT and CSC-like properties of MDA-MB-231 and SUM159 cells, assessed by western blot. (H) RT-qPCR analysis of EMT and CSC marker expression in MDA-MB-231 and SUM159 cells co-cultured with M0 or M2 macrophages. (I-K) TNBC cells were tested for cell migration, invasion, and stemness (magnification, 100×, scale bar=100 μm) utilizing the wound healing assay, Transwell invasion assay, and microsphere formation experiment, in that order. *P < 0.05, **P < 0.01, ***P < 0.001.

Tumor cells and other constituents within the TME are capable of steering macrophage polarization towards the M2 phenotype, which confers upon them the ability to mediate immunosuppression and augment tumor-promoting activities. We first generated an external model to mimic TAMs, as illustrated in Fig. 2A. The induced M2 macrophages displayed enhanced activity and formed a lot of dendritic-like branches. Flow cytometry analysis revealed increased CD163 expression in M2 macrophages (Fig. 2B). RT-qPCR demonstrated upregulation of M2-associated markers, including CD163, TGF-β, and IL-10, in the induced cells relative to the THP-1 cells. Conversely, M1 macrophage markers such as TNF-α, HLA-DR, and iNOS exhibited reduced expression levels (Fig. 2C). Western blot analysis corroborated these findings, with increased Arg1 protein observed in M2 macrophages (Fig. S1A). After co-culturing with TNBC cells (MDA-MB-231 and SUM159), M0 macrophages were also polarized to the M2 phenotype, as evidenced by the maintenance of their characteristic morphology post-co-culture (Fig. 2D). The expression of pertinent markers was further validated by RT-qPCR (Fig. 2E) and western blot (Fig. 2F). Collectively, the above results indicate that TNBC cells mostly induced M2 polarization of macrophages.

To investigate the relationship between EMT and the generation of CSC characteristics, we examined the expression of EMT and CSC markers in the TNBC cell lines MDA-MB-231 and SUM159 following co-cultured with M0 or M2 macrophages. Our results revealed a decrease in the epithelial marker E-cadherin and an increase in mesenchymal markers N-cadherin and Snail, as determined by western blot and RT-qPCR. The levels of CSC-associated transcription factors, including SOX2, Nanog, and OCT4, were elevated in both cell lines, suggesting that the presence of M2 macrophages may promote EMT and enhance the stem-like properties of TNBC cells (Fig. 2G and H). Following 48 hours of co-culture with M2 macrophages, we assessed the migratory and invasive capacities of the MDA-MB-231 and SUM159 cell lines using wound-healing and Transwell assays. Notably, cells co-cultured with M2 macrophages demonstrated accelerated wound closure, a phenotype that was corroborated by the Transwell assay results (Fig. 2I, J, S1B, and C). The impact of M0- and M2-macrophage-conditioned media (M0-CM and M2-CM) on the stemness of these cells was evaluated by culturing MDA-MB-231 and SUM159 in ultra-low attachment plates. The M2-CM treatment markedly promoted the formation of spheres (Fig. 2K), indicative of an enhanced stem cell-like phenotype. In summary, these findings suggest that TAMs, particularly those polarized towards the M2 phenotype, can induce EMT and significantly augment the migratory, invasive, and CSC properties of TNBC cells.

### TAM-derived eNAMPT promoted malignant behavior in TNBC cells

**Figure 3:**
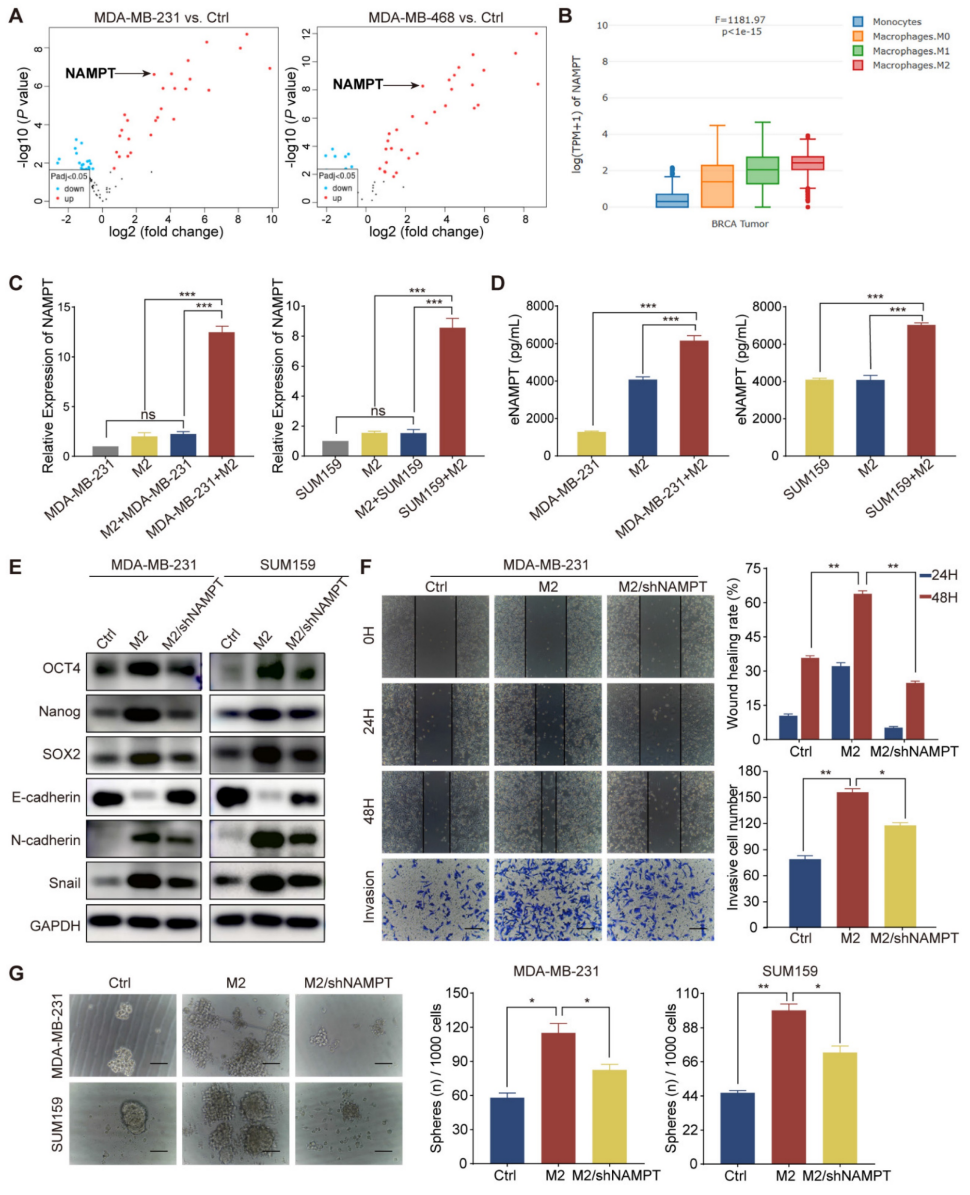
** TAM-derived eNAMPT promotes TNBC malignancy.** (A) The volcano plot illustrates differential gene expression in macrophages polarized by TNBC cells compared to those cultured in isolation, with upregulated and downregulated genes indicated. (B) NAMPT expression is shown to vary across different macrophage types within breast cancer tissues. (C-D) RT-qPCR and ELISA were used to measure NAMPT levels in MDA-MB-231 and SUM159 cells and M2 macrophages under co-culture and monoculture conditions. mRNA was extracted from MDA-MB-231 cells in the M2+MDA-MB-231 group to assess the influence of M2 macrophages on tumor cell NAMPT expression. In the MDA-MB-231+M2 group, mRNA was extracted from M2 macrophages to evaluate the influence of tumor cells on macrophage NAMPT expression. (E) Western blot analysis evaluates the expression of EMT and CSC markers in MDA-MB-231 and SUM159 cells cultured alone, co-cultured with M2 macrophages, or co-cultured with M2 macrophages in which eNAMPT expression was silenced. (F) Wound-healing and Transwell assays assess the migratory and invasive capabilities of MDA-MB-231 cells (magnification, 100×, scale bar=100 μm). (G) The microsphere formation assay estimates the stemness of TNBC cells in response to the presence or absence of NAMPT from M2 macrophages (magnification, 100×, scale bar=100 μm). *P < 0.05, **P < 0.01, ***P < 0.001, ns means not significant.

TAMs interact with TNBC cells through complex signaling pathways, influencing cellular processes such as migration, invasion, and the acquisition of aggressive phenotypes by modulating the secretion of cytokines, growth factors, chemokines, and proteases 22, 23. To elucidate the regulatory mechanisms by which M2 macrophages influence the biology of TNBC cells, we initiated our investigation with a bioinformatics analysis of the GSE92927 dataset. This dataset encompasses gene expression profiles from macrophages derived from human monocytes under two conditions: in isolation and in co-culture with TNBC cells, specifically MDA-MB-231 and MDA-MB-468. Through this analysis, we identified a distinct set of 35 differentially expressed genes within M2 macrophages polarized by TNBC cells (Fig. S2A). NAMPT was found to be significantly upregulated in M2 macrophages in response to TNBC cell polarization compared to control macrophages cultured in isolation (Fig. 3A). Our analysis utilizing the GEPIA2021 database revealed a significant upregulation of NAMPT expression in breast cancer tissues, particularly within the M2 macrophage subset, as compared to M1/M0 macrophages and monocytes (Fig. 3B). Further examination of NAMPT expression in breast cancer tissues through TIMER, TISIDB, and HPA databases demonstrated a positive association between NAMPT levels and macrophage immune enrichment, as well as a correlation with the M2 macrophage markers CD163 and CD206 (Fig. S2B, C, and D). NAMPT expression was substantially elevated in TNBC tissues relative to normal or other breast cancer subtypes (*P* = 0.003, Fig. S2E). Protein expression analysis via the HPA database, corroborated by immunohistochemical staining, confirmed these findings, highlighting the elevated presence of NAMPT in breast cancer tissues (Fig. S2F). Consistent with this, NAMPT mRNA levels were significantly higher in TNBC cell lines BT-549, MDA-MB-436, and MDA-MB-231 compared to non-TNBC cell lines such as MCF7, T47D, and SKBR3 (Fig. S2G). RT-qPCR analysis indicated that M2 macrophages exhibited increased NAMPT mRNA levels, which were further induced upon co-cultured with TNBC cell lines MDA-MB-231 and SUM159, without a significant increase in the TNBC cell lines themselves (Fig. 3C). ELISA experiments substantiated these observations, showing a marked increase in eNAMPT protein levels in supernatants from M2 macrophage and TNBC cell co-cultures compared to individual cultures (Fig. 3D). These data collectively suggest that TAMs may exert their effects on TNBC cells, in part, through the secretion of eNAMPT, thereby influencing the tumor's biological behavior.

To investigate the role of eNAMPT in the behavior of TNBC cells, we utilized a shRNA lentivirus to knock down eNAMPT expression in THP-1 cells, subsequent to which the cells were induced to adopt the M2 macrophage phenotype. Subsequent western blot analysis revealed that NAMPT suppression resulted in a downregulation of the epithelial marker E-cadherin and an upregulation of mesenchymal markers N-cadherin and Snail, as well as increased levels of stemness-associated transcription factors SOX2, Nanog, and OCT4 in both MDA-MB-231 and SUM159 cell lines (Fig. 3E). The impact of eNAMPT silencing on cancer cell migration and invasion was investigated, demonstrating that NAMPT suppression mitigated these capabilities, which are typically enhanced by M2 macrophage influence (Fig. 3F and Fig. S2H). Sphere formation assays further confirmed that NAMPT knockdown led to a reduction in both the size and number of tumor spheres, indicative of diminished CSC-like properties (Fig. 3G). Collectively, these findings indicate that eNAMPT, secreted by TAMs, is a critical factor in promoting the aggressive phenotype of TNBC cells.

### TAM-derived eNAMPT enhanced EMT and CSC-like properties via binding to CCR5

**Figure 4:**
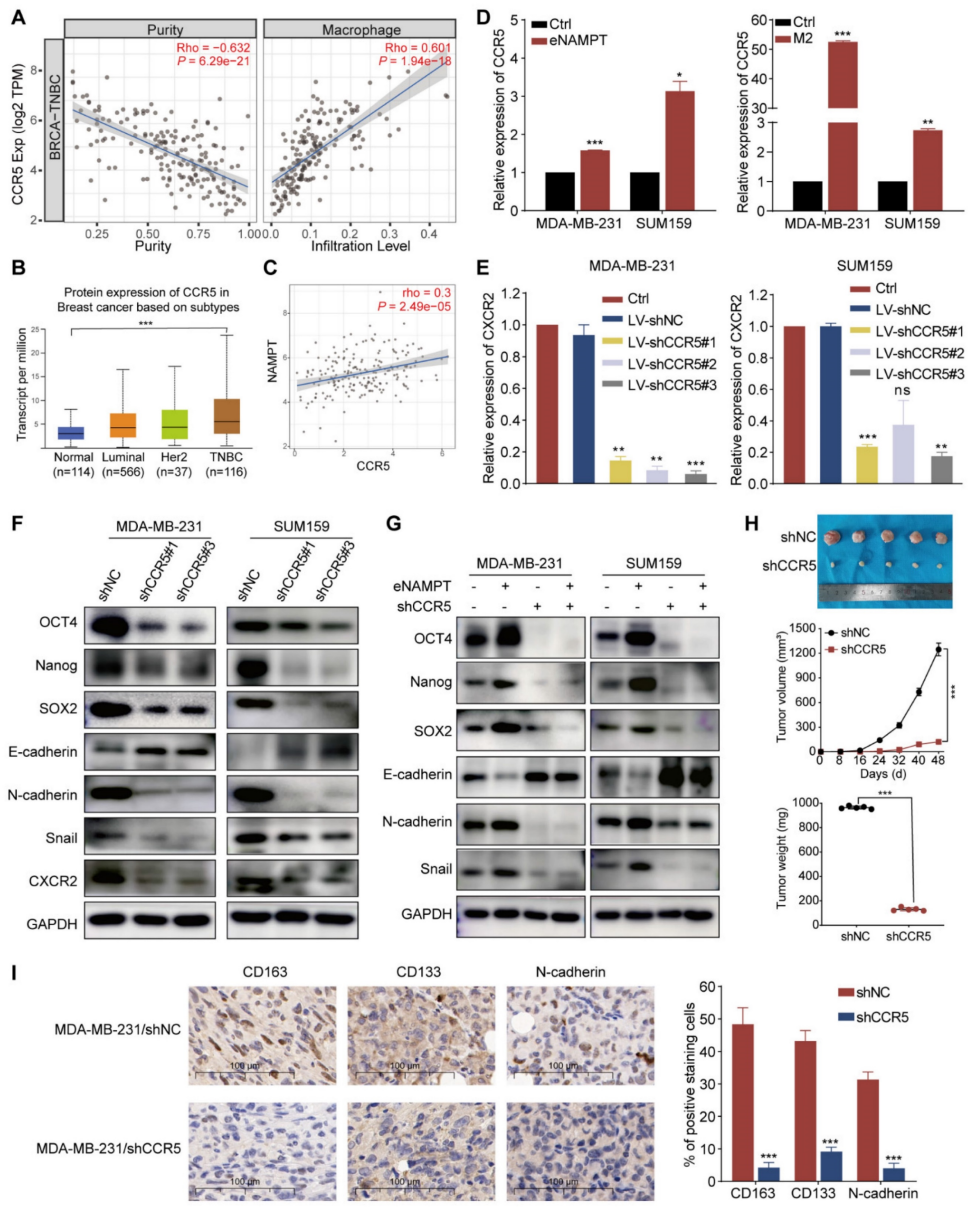
** eNAMPT promotes EMT via CCR5 engagement in TNBC.** (A) Analysis demonstrates a positive correlation between CCR5 expression and macrophage infiltration within the TNBC microenvironment. (B) Comparative assessment of CCR5 expression across normal tissues and various breast cancer subtypes. (C) Correlation analysis reveals a positive association between CCR5 and NAMPT expression specifically in TNBC tissues. (D) eNAMPT (20 nM) stimulation or co-cultured with M2 macrophages upregulates CCR5 expression in MDA-MB-231 and SUM159 TNBC cells. (E) RT-qPCR confirms significant downregulation of CCR5 following transfection with Lentivirus-shCXCR2 in MDA-MB-231 and SUM159 cells. (F) Western blot analysis quantifies the expression of EMT and CSC markers in CCR5-silenced TNBC cells (MDA-MB-231 and SUM159) relative to control cells. (G) Western blot examines the effects of eNAMPT or shCCR5 on the protein expression in MDA-MB-231 and SUM159 cells. (H) Tumor volume measurements after 8 weeks in tumor-bearing mice show that CCR5 knockdown results in significantly smaller tumors compared to the control group (*n*=5). (I) Immunohistochemical (IHC) assays indicate that CCR5 silencing leads to reduced expression of CD163, CD133, and N-cadherin in tumor tissues (*n*=5, magnification, 400×, scale bar=100 μm). *P < 0.05, **P < 0.01, ***P < 0.001, ns means not significant.

Prior research has established eNAMPT as a pivotal regulator in innate immunity and inflammation, with its role in cancer progression linked to interactions with Toll-like receptor 4 (TLR4) and the chemokine receptor CCR5 24. In this segment of our investigation, we aimed to elucidate the mechanisms by which eNAMPT, secreted by TAMs, modulates the biology of TNBC cells, with a particular focus on the potential involvement of CCR5. We initiated our analysis by examining the TCGA database to assess CCR5 expression within breast cancer tissues and explore the influence of CCR5 on the breast cancer microenvironment. Our findings revealed a significant positive correlation between CCR5 expression and macrophage immune infiltration within the TNBC microenvironment (Fig. 4A and S3A). When examining CCR5 expression in breast cancer tissues, we observed the highest levels in TNBC, surpassing those in normal tissues or other breast cancer subtypes (Fig. 4B and S3B). Intriguingly, a positive correlation was identified between CCR5 and NAMPT expression specifically within TNBC tissues (Fig. 4C and S3C). These results suggest that the interaction between eNAMPT and CCR5 may play a crucial role in the modulation of TNBC cell behavior by TAMs.

To further investigate the role of TAM-derived eNAMPT in inducing EMT and CSC-like characteristics in TNBC cells, we treated TNBC cell lines with exogenous eNAMPT or M2-CM. Our results showed a significant increase in CCR5 expression in TNBC cells in response to eNAMPT (Fig. 4D). Subsequently, CCR5 was knocked down in MDA-MB-231 and SUM159 cells using shRNA, and the efficiency was confirmed by RT-qPCR (Fig. 4E). Western blot analysis of stably transfected cell lines revealed that CCR5 knockdown led to an increase in E-cadherin expression and a decrease in the expression of SOX2, Nanog, OCT4, N-cadherin, and Snail, indicating a reversal of EMT and CSC properties (Fig. 4F). Exogenous eNAMPT increased the expression of CSC- and mesenchymal-related proteins, but this effect was abolished upon CCR5 blockade (Fig. 4G). Additionally, eNAMPT-enhanced migration and invasion of TNBC cells were mitigated by CCR5 inhibition (Fig. S3D and E), and the increase in sphere formation induced by eNAMPT was also reversed by CCR5 knockdown (Fig. S3F). To validate the *in vivo* relevance of the eNAMPT/CCR5 signaling axis, orthotopic allograft models were established. Tumor volume and weight were significantly reduced in the MDA-MB-231-shCCR5 group compared to the MDA-MB-231-shNC control group (Fig. 4H). CCR5 silencing resulted in decreased protein levels of CD133, Nanog, N-cadherin, and CD163 *in vivo* (Fig. 4I). Collectively, these findings underscore the critical role of eNAMPT/CCR5 signaling in the promotion of aggressive and stemness features in TNBC.

### TAMs contributed to EMT and CSC-like properties via STAT3 phosphorylation and acetylation

**Figure 5:**
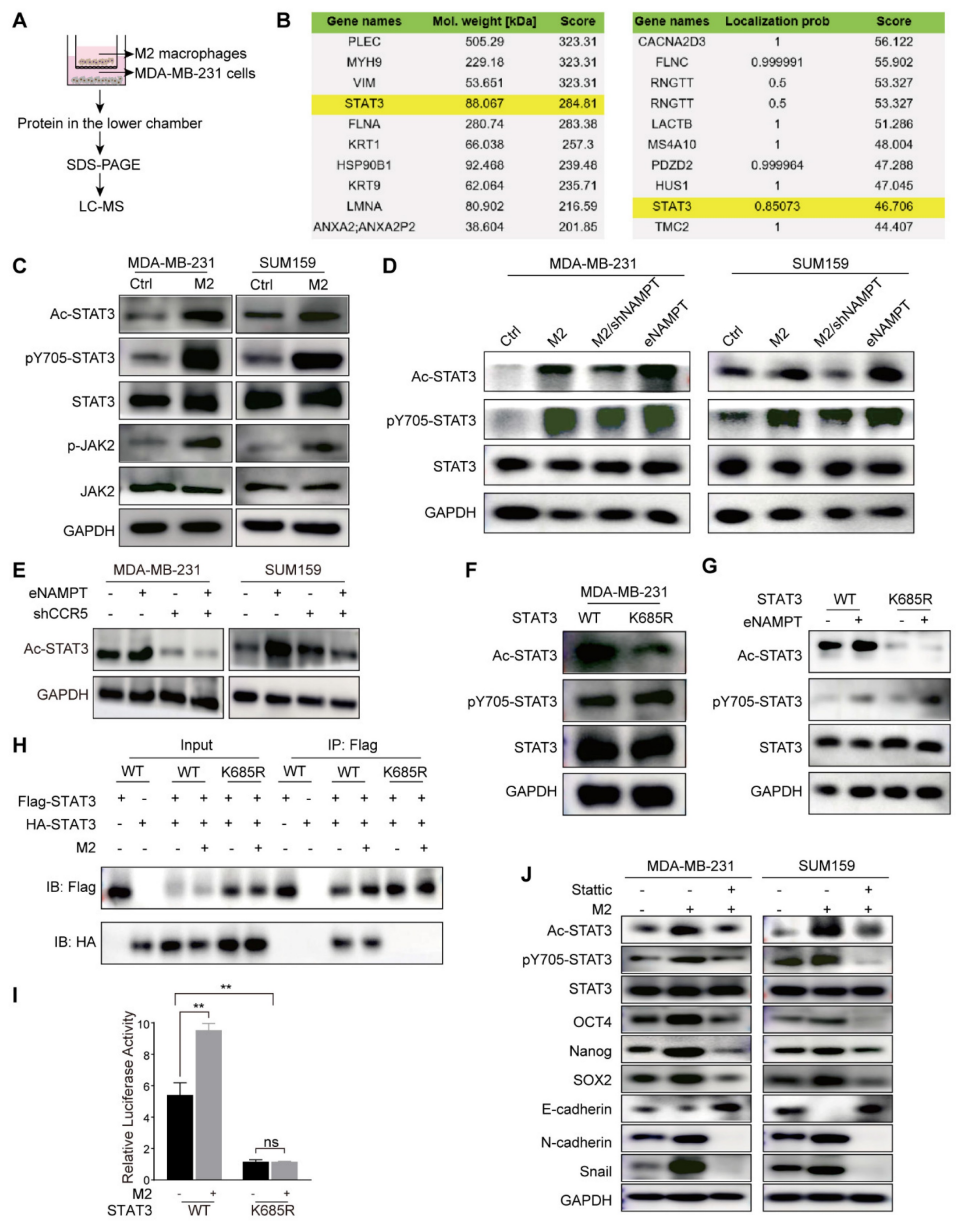
** STAT3 phosphorylation and acetylation contribute to TAM-induced EMT and malignant behavior of TNBC cells.** (A) The flowchart presents the workflow of protein mass spectrometry analysis. (B) Mass spectrometry identified the top 10 total and acetylated non-histone proteins, ranked by protein score and localization probability. (C) Co-cultured with M2 macrophages activates STAT3 phosphorylation and acetylation in MDA-MB-231 and SUM159 cells. (D) Western blot analysis demonstrates the effects of M2 macrophages and eNAMPT on STAT3 signaling, in the presence or absence of shNAMPT or eNAMPT. (E) Western blot analysis detects the impact of eNAMPT/CCR5 on STAT3 acetylation in TNBC cells. (F) STAT3^K685R^ mutation leads to STAT3 deacetylation in MDA-MB-231. (G) Wild-type STAT3 exhibits phosphorylation and acetylation in response to eNAMPT treatment, while the mutant shows only phosphorylated-STAT3 activation in MDA-MB-231 cells. (H) STAT3^K685R^ prevents the formation of STAT3 dimers. (I) Relative luciferase activity of Wild-type STAT3 and STAT3^K685R^ in MDA-MB-231 cells co-cultured with M2 macrophages. (J) Western blot examination of TNBC (MDA-MB-231 and SUM159) cells grown either alone or in combination with M2 macrophages, with or without Stattic (10 μM). **P < 0.01, ns means not significant.

To elucidate the precise mechanisms underlying the promotion of TNBC cell migration, invasion, and stemness by TAMs through the secretion of eNAMPT and its interaction with CCR5, we established a co-culture system of M2 macrophages and MDA-MB-231 cell, for a duration of 48 hours. Following this incubation period, total protein extracts were subjected to mass spectrometry analysis to identify potential signaling molecules (experimental workflow depicted in Fig. 5A). The mass spectrometry data revealed the presence of STAT3 among the co-cultured proteins, with Fig. 5B illustrating the top 10 protein molecules ranked by their respective scores. STAT family proteins are activated through tyrosine phosphorylation at specific sites, which triggers the formation of homodimers or heterotrimers with other transcription factors 25. However, evidence suggests that unphosphorylated or tyrosine-mutated STAT proteins retain the capacity to form dimers and activate transcription, implying the existence of alternative regulatory mechanisms that stabilize STAT dimer formation 26. Evidence has highlighted lysine acetylation as an additional modulatory mechanism that can control STAT3 protein activity and the CSC-like properties of various cancers 27. To this end, proteins derived from co-cultured TNBC cells and M2 macrophages were analyzed by acetylation mass spectrometry. The data revealed that STAT3 was subject to acetylation in TNBC cells following co-culture with M2 macrophages (Fig. 5B).

Treatment with both TAM supernatant and eNAMPT upregulated phospho-JAK2, phospho-STAT3, and acetyl-STAT3 in MDA-MB-231 and SUM159 cells (Fig. 5C and D). To further assess the role that this signaling pathway plays in TAM-induced stemness in TNBC, M2 macrophages with stable NAMPT knockdown were produced. The results showed that silencing NAMPT downregulated phospho-STAT3 and acetyl-STAT3. Treatment with eNAMPT promoted the expression of phospho-STAT3 and acetyl-STAT3 (Fig. 5D). The addition of eNAMPT led to a significant increase in Ac-STAT3 levels in MDA-MB-231 and SUM159 cell lines. Conversely, the knockdown of CCR5 resulted in a notable reduction in Ac-STAT3 expression. Importantly, the suppression of Ac-STAT3 induced by CCR5 knockdown was not reversed by the addition of exogenous eNAMPT to the CCR5-knockdown TNBC cells (Fig. 5E). These results collectively indicate that TAMs facilitate the phosphorylation and acetylation of STAT3 through the secretion of eNAMPT, which engages with CCR5 on the surface of TNBC cells.

To explore the consequences of STAT3 acetylation on its transcriptional activity and its role in tumor progression, we generated MDA-MB-231 cells harboring a mutant form of STAT3 (STAT3^K685R^), wherein Lys685 of STAT3 was changed to Arg. After induction with M2-CM, STAT3^K685R^ was not acetylated compared with wild-type (WT) STAT3, but the tyrosine of both WT STAT3 and STAT3^K685R^ were phosphorylated (Fig. 5F). Consistently, similar modifications were observed in response to eNAMPT (Fig. 5G). We transfected MDA-MB-231 cells with both Flag- and HA-tagged STAT3. We used western blot on anti-Flag immunoprecipitates from extracts of these transfected cells to detect associations between these two forms of STAT3, with anti-HA. We found that in cells transfected with Flag-tagged and HA-tagged wild-type (WT) STAT3, co-immunoprecipitation revealed an association between these two forms of STAT3, which was stabilized by treatment with M2-CM. However, the association between HA-STAT3^K685R^ and Flag-STAT3^K685R^ was undetectable (Fig. 5H). This signaled acetylation of Lys685 is crucial for STAT3 dimer formation. We then conducted a STAT3-dependent luciferase activity test which showed that only WT STAT3, not STAT3^K685R^, could restore M2 macrophages response in MDA-MB-231 cells (Fig. 5I). These findings indicate that STAT3 acetylation affects its transcriptional activity by modulating dimer formation. Stattic, a STAT3 inhibitor, which resulted in a significant blockage of M2 macrophage-induced expression of p-STAT3 and Ac-STAT3, and decreased EMT and CSC-like properties in MDA-MB-231 and SUM159 cells (Fig. 5J). Moreover, Stattic inhibited the M2 macrophage-enhanced migration, invasion, and microsphere formation of TNBC cells (Fig. S4A and B). The results concluded that eNAMPT, which is produced by M2 macrophages, may enhance TNBC cell motility, invasion, and CSC-like characteristics through the STAT3 signaling pathway.

### Acetylation of STAT3 at Lys685 regulated DIRAS2 gene promoter methylation

**Figure 6:**
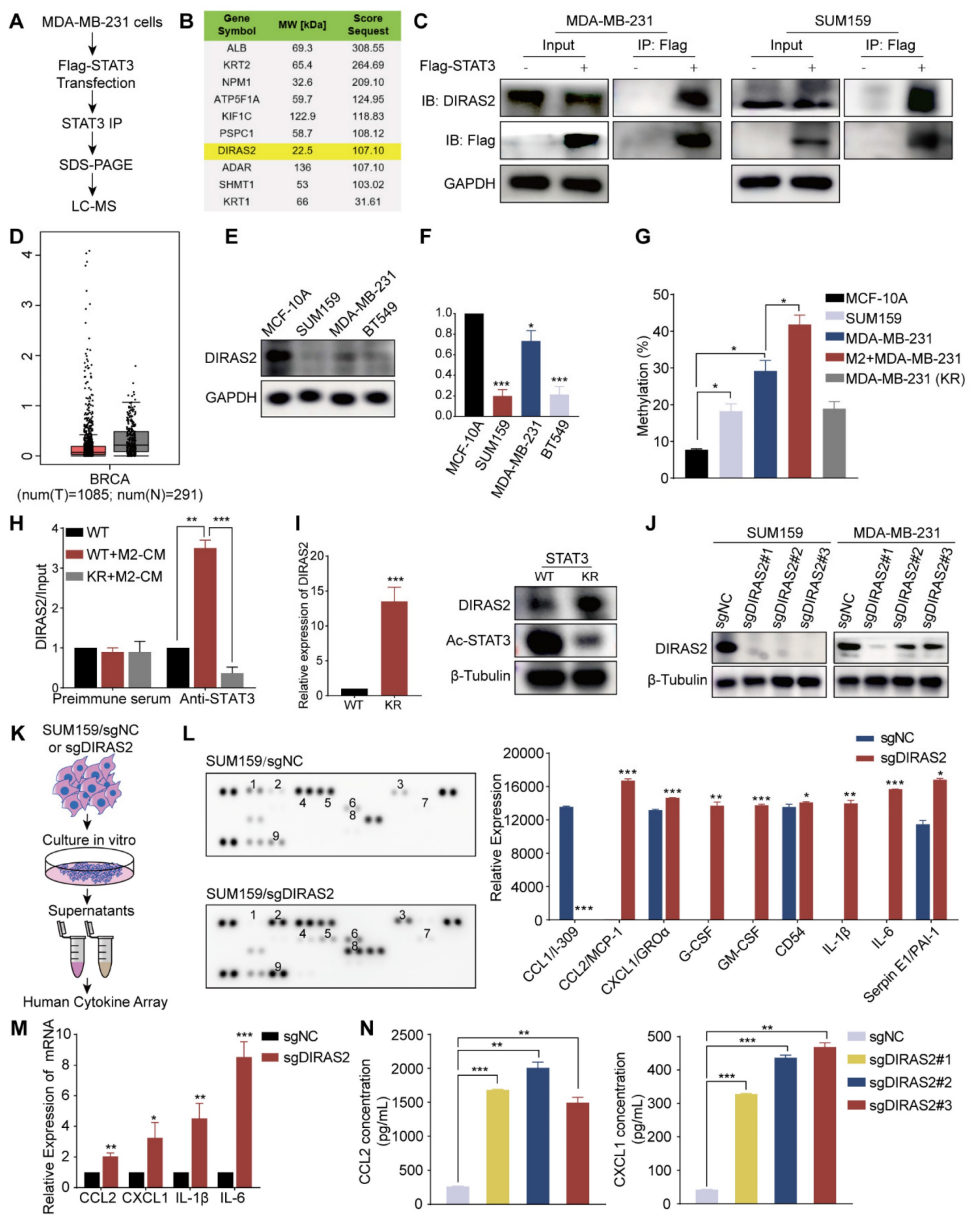
** Activated STAT3 acetylation leads to DIRAS2 gene promoter methylation.** (A) The flowchart outlines the process of mass spectrometry analysis for immunoprecipitated proteins. (B) Mass spectrometry identifies the top 10 proteins interacting with STAT3, ranked by protein score and localization probability. (C) The interaction between STAT3 and DIRAS2 proteins is confirmed in MDA-MB-231 and SUM159 cells. (D) DIRAS2 expression is significantly lower in breast cancer tissues compared to normal tissues. (E-F) Western blot and RT-qPCR analyze DIRAS2 expression in TNBC cells (MDA-MB-231, SUM159, and BT549) and normal breast epithelial cells (MCF-10A). (G) Pyrosequencing analysis detects DIRAS2 promoter methylation in normal breast epithelial cells (MCF-10A), TNBC cells (MDA-MB-231 and SUM159), MDA-MB-231 cells co-cultured with M2 macrophages, and MDA-MB-231 cells containing the STAT3^K685R^ mutant. (H) ChIP analysis assesses the binding of STAT3 to DIRAS2 promoters. (I) mRNA and protein levels of DIRAS2 in MDA-MB-231 cells transfected with WT or KR STAT3 are measured. (J) Western blot confirms the loss of DIRAS2 expression in TNBC cells (MDA-MB-231 and SUM159) following CRISPR-mediated gene editing. (K) Schematic illustration of sample preparation for cytokine array analysis. (L) Human cytokine array analysis reveals differentially expressed cytokines in DIRAS2-deficient SUM159 cells. (M) RT-qPCR shows increased expression of CCL2, CXCL1, IL-1β, and IL-6 in SUM159 cells after DIRAS2 knockdown. (N) ELISA confirms the increased secretion of CCL2 and CXCL1 in SUM159 cells following DIRAS2 loss. *P < 0.05, **P < 0.01, ***P < 0.001.

To delineate the molecular interactions involving acetylated STAT3 and to ascertain its functional role in TNBC, we employed a targeted proteomic approach. Enrichment of FLAG-tagged STAT3 protein was conducted, followed by LC-MS analysis to identify interacting proteins (Fig. 6A). Among the identified proteins, DIRAS2 was revealed as a potential interactor with STAT3 (Fig. 6B). To substantiate this interaction, Co-IP assays were performed on the TNBC cell lines MDA-MB-231 and SUM159. The results confirmed a physical association between STAT3 and DIRAS2, thereby implicating STAT3 in the regulation of DIRAS2 protein complex formation (Fig. 6C). To explore the role of DIRAS2 in breast cancer, a comprehensive literature review and bioinformatics analysis using the GEPIA database were conducted, suggesting that DIRAS2 functions as a tumor suppressor protein in breast cancer (Fig. 6D). Further assessment of DIRAS2 mRNA and protein levels across a panel of TNBC cell lines, including MDA-MB-231, SUM159, and BT549, corroborated the tissue staining results, showing a significant downregulation of DIRAS2 in TNBC cells compared to normal breast cell lines (Fig. 6E and F). These findings collectively implicate DIRAS2 as a putative tumor suppressor whose loss may contribute to the aggressive phenotype of TNBC.

To elucidate the epigenetic mechanisms by which acetylated STAT3 modulates tumor-suppressor gene expression in TNBC, we hypothesized that this modification might enhance CpG island methylation, thereby downregulating the expression of such genes. Focusing on the DIRAS2 promoter, we explored the association between acetylated STAT3 binding and methylation status across different cell models. Our investigation began with an analysis of methylation CpG island proportions in non-malignant MCF-10A cells and TNBC cell lines MDA-MB-231 and SUM159. The methylation CpG island proportion in MCF-10A cells was 7.7 ± 0.3%, whereas in MDA-MB-231 and SUM159 cells, it was 29.2 ± 2.8% and 18.2 ± 2.1% (*P* < 0.05), as depicted in Fig. 6G. The results indicate that there was either partial methylation or hypermethylation of the CpG island in TNBC cells.

Further experiments in MDA-MB-231 cells revealed that acetylated STAT3, induced by M2 macrophages, was capable of promoting DIRAS2 promoter methylation, an effect that was mitigated by the K685R mutation in STAT3, resulting in decreased acetylation levels (Fig. 6G). Employing ChIP assays, we confirmed that acetylated STAT3 preferentially bound to the DIRAS2 promoter in wild-type MDA-MB-231 cells compared to the STAT3^K685R^ mutant cell line (Fig. 6H). The transfection of MDA-MB-231 cells with the STAT3^K685R^ mutant led to an increase in DIRAS2 expression at both the RNA and protein levels, which corresponded with reduced STAT3 acetylation (Fig. 6I). From these results, we concluded that acetylated STAT3 accelerates the methylation of the DIRAS2 promoter, playing a pivotal role in the gene silencing and thereby contributing to the pathogenesis of TNBC.

### Ablation of DIRAS2 in TNBC cells enhanced CCL2 secretion and macrophage recruitment

Cancer cells with deletion of tumor suppressor genes can escape the immune response 15. The above section reveals that the DIRAS2 gene functions as a tumor suppressor in TNBC; thus, we investigated the association of immune infiltration with DIRAS2 expression in TNBC. Many chemokines, cytokines, and growth factors act as messengers between tumor cells and immune cells 28. In order to determine how DIRAS2 affected the immune response against tumors, we generated TNBC cells lacking DIRAS2 expression (Fig. 6J), cultured them or a control group, and collected corresponding supernatants for subsequent experiments (Fig. 6K). Human cytokine array analysis showed significant alterations in the secretion patterns of DIRAS2-deficient cells (Fig. 6L). Several cytokines, including GM-CSF, IL-1β, CCL2, and IL-6, showed significant increases in the supernatants of DIRAS2-depleted SUM159 cells, indicating a potential link between DIRAS2 loss and immune modulation. Using three DIRAS2 sgRNAs, we validated that loss of DIRAS2 increases the mRNA level of these top genes in SUM159 cells (Fig. 6M). ELISA also showed increased CCL2 and CXCL1 secretion (Fig. 6N). The observed upregulation of CCL2 is particularly noteworthy, as it is a known mediator of myeloid-derived suppressor cell (MDSC) and macrophage recruitment and polarization within the TME. These findings suggest that the ablation of DIRAS2 in TNBC cells may enhance the recruitment of immunosuppressive cells, thereby facilitating immune evasion and potentially contributing to tumor progression.

**Figure 7:**
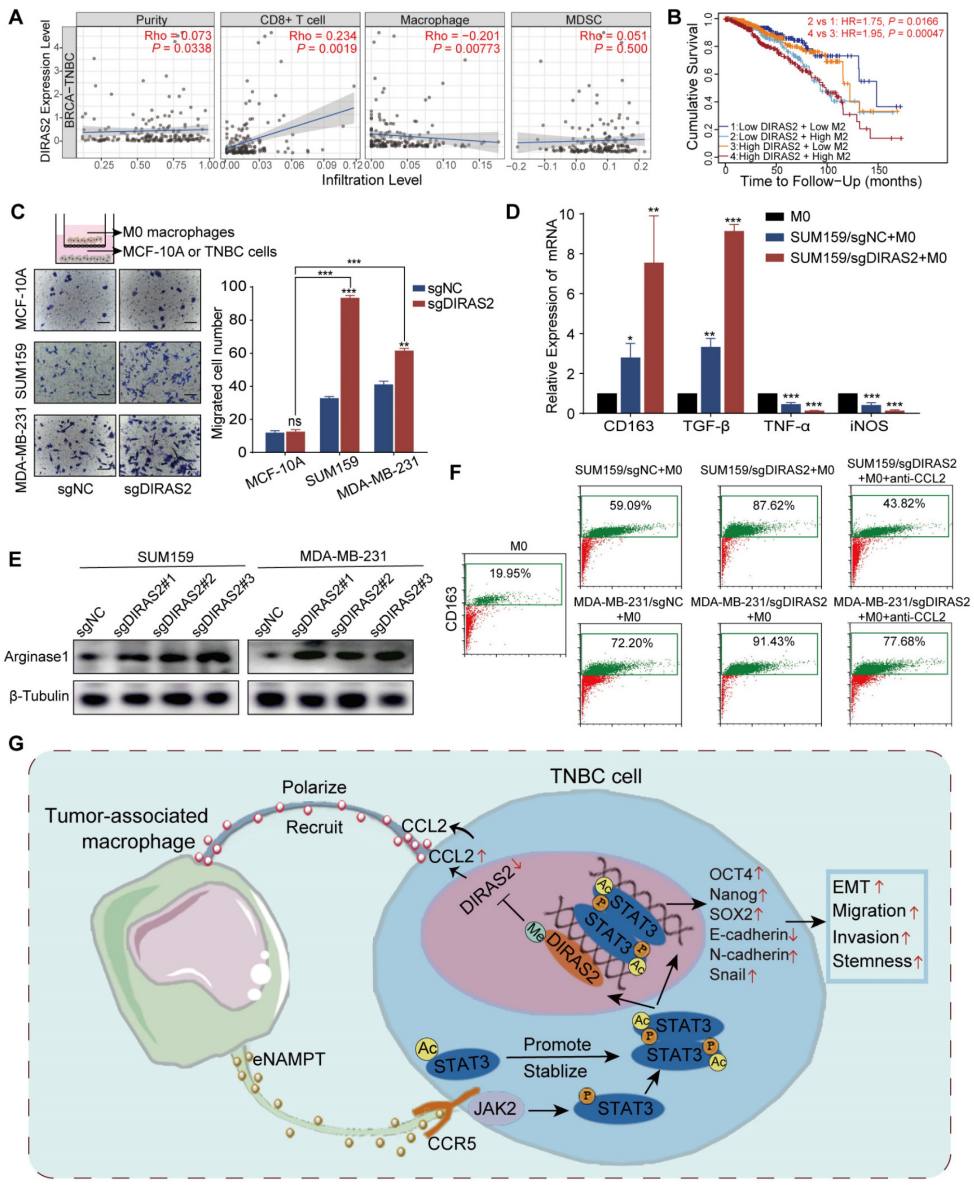
** DIRAS2 ablation in TNBC cells enhances CCL2 secretion and macrophage recruitment.** (A) Analysis of immune infiltration indicates a correlation between DIRAS2 expression in TNBC and the infiltration of immune cells, including CD8^+^ T cells, macrophages, and MDSC. (B) A Kaplan-Meier curve demonstrates the association between DIRAS2 expression, M2 macrophage infiltration, and overall survival in TNBC patients. (C) A schematic diagram illustrates the setup of a transwell system with M0 macrophages in the upper chamber and either normal breast epithelial cells (MCF-10A) or TNBC cells (MDA-MB-231 and SUM159) in the lower chamber. The migration of M0 macrophages was quantified 24 hours post-seeding. (D) RT-qPCR analysis examines the expression of M2 (CD163, TGF-β) and M1 (TNF-α, iNOS) macrophage markers in M0 macrophages, M0 macrophages co-cultured with SUM159/sgNC, and M0 macrophages co-cultured with SUM159/sgDIRAS2. (E) Western blot analysis assesses Arginase 1 expression in M0 macrophages cultured alone, M0 macrophages co-cultured with SUM159/sgNC, and M0 macrophages co-cultured with SUM159/sgDIRAS2. (F) Flow cytometry analysis measures CD163 expression in M0 macrophages, M0 macrophages co-cultured with SUM159/sgNC, M0 macrophages co-cultured with SUM159/sgDIRAS2, and M0 macrophages co-cultured with CCL2-depleted SUM159/sgDIRAS2. (G) A diagrammatic representation summarizes the interplay between TAMs and cancer cells in the tumor microenvironment. *P < 0.05, **P < 0.01, ***P < 0.001, ns means not significant.

In TNBC patients, DIRAS2 expression levels exhibited a positive correlation with CD8^+^ T cell infiltration and a negative correlation with macrophage infiltration, while DIRAS2 expression was not significantly associated with MDSC infiltration (Fig. 7A). Importantly, our research has also revealed a significant association between TNBC patient survival rates and the levels of DIRAS2 expression and M2 macrophage infiltration (Fig. 7B). Transwell assays have demonstrated that the depletion of DIRAS2 in TNBC cells enhances macrophage chemotaxis, an effect not observed with normal mammary epithelial cells (Fig. 7C). These results imply that DIRAS2 depletion affects macrophage chemotaxis and the secretome of cancer cells. Co-culture experiments with DIRAS2-depleted TNBC cells and THP-1-derived M0 macrophages have shown a shift towards an M2 phenotype, as evidenced by upregulation of M2-associated markers (CD163 and TGF-β) and downregulation of M1 markers (TNF-α and iNOS) (Fig. 7D). Western blot verified that M2 maker ARG1 was upregulated (Fig. 7E). This phenotype modulation was abrogated by the addition of a CCL2-neutralizing antibody, implicating CCL2 as a key mediator of macrophage polarization in this context (Fig. 7F). Diagrammatic representation of the interaction between TNBC cells and TAMs was shown in Fig. 7G. In the TNBC microenvironment, TAM-secreted eNAMPT interacts with the CCR5 receptor on the surface of cancer cells. This interaction triggers the phosphorylation and acetylation of STAT3, leading to the formation of homologous or heterodimers. These dimers translocate to the nucleus, where they regulate gene expression and facilitate the EMT program, enhancing the migratory, invasive, and metastatic capabilities of TNBC cells. The methylation of the DIRAS2 gene promoter, which results in its loss, is encouraged by STAT3 acetylation at Lys685. The loss of DIRAS2 is implicated in increased secretion of CCL2 by TAM-educated TNBC cells, which in turn, amplifies macrophage recruitment and contributes to the immunosuppressive and protumorigenic characteristics of the TNBC microenvironment.

## Discussion

### Main interpretation

Neoplasms remain the main killer worldwide 29-32. TNBC is characterized by aggressive behavior, poor prognosis, and a dearth of effective treatment strategies. TAMs, as vital components of the TME, are critically involved in cancer progression. Macrophages present in the TME can impact the differentiation and phenotype of tumor cells, encouraging tumor growth, metastasis, and resistance to chemotherapy 17, 33, 34. While the interaction between TAMs and EMT in TNBC has been extensively studied, the mechanisms by which TAMs affect CSCs in TNBC are not well understood. Our study uncovered a distinct cytokine-mediated communication between TAMs and TNBC cells, activating the STAT3 signaling pathway and driving TAM-induced EMT and the acquisition of stem-like properties in TNBC. We observed that higher infiltration of CD163-positive TAMs in TNBC tissues correlated with poorer patient outcomes. We demonstrated that eNAMPT from TAMs engages with CCR5 on TNBC cells, triggering STAT3 phosphorylation and acetylation. This event leads to the downregulation of DIRAS2, which in turn stimulates CCL2 secretion by TNBC cells. The secretion of CCL2 subsequently facilitates EMT, enhances cellular migration and invasion, and induces stemness in TNBC cells, as well as macrophage recruitment.

The TME is a complex ecosystem comprising not only tumor cells but also a variety of stromal cells, including fibroblasts, immune cells, and inflammatory cells, as well as the extracellular matrix, microvessels, and infiltrating biomolecules 35. The interaction between different cells or between cells and factors alters the function of tumor cells and immune cells, forms a dynamic and complex tumor immunological microenvironment, and encourages the growth, invasion, and metastasis of tumor cells 35, 36. TAMs, in particular, play a critical role in sculpting the inflammatory TME and have been implicated in the progression of various cancers. Our study provides evidence that M2 macrophages, when co-cultured with TNBC cells, enhance the invasive and migratory capabilities of these cells, as well as their CSC-like properties. This is in line with the established notion that TAMs, characterized by their M2 phenotype, secrete a plethora of cytokines, chemokines, and proteases, which activate signaling pathways that contribute to CSC formation and maintenance, thereby exacerbating tumor progression and resistance to chemotherapy 37, 38. For example, TAMs have been shown to regulate the IL-6/JAK2/STAT3/miR-506-3p/FoxQ1 axis, which enhances colorectal cancer (CRC) motility and invasion through the induction of EMT and upregulation of CCL2, promoting macrophage recruitment 39. IL-15Rα^+^ TAMs have been reported to limit the recruitment of CD8^+^ T lymphocytes by reducing CX3CL1 expression in breast cancer cells and releasing the IL-15/IL-15Rα complex (IL-15Rc) 40. These findings underscore the significance of cytokine and chemokine signaling pathways in the crosstalk between tumor cells and macrophages. To further explore this interaction, we conducted cytokine analysis on the supernatants of polarized macrophages, revealing that several macrophage-derived cytokines, including eNAMPT, CCL3/CCL4, G-CSF, GM-CSF, CD54, and IL-6, were differentially regulated following exposure to TNBC cell-conditioned medium. This suggests a potential feedback loop between TAMs and TNBC cells, where TAM-derived factors can modulate the behavior of TNBC cells, and vice versa.

Extracellular nicotinamide phosphoribosyltransferase (eNAMPT), a secreted form of the intracellular enzyme NAMPT, is produced by a variety of cell types, including tumor cells, immune cells, adipocytes, fibroblasts, and endothelial cells. It has been consistently reported to be significantly elevated in the plasma of patients with inflammatory diseases and cancer 41-43. While NAMPT expression is notably increased in both tumor and immune cells and can be released in a Brefeldin A-dependent or independent manner, the precise cellular source remains elusive 44. In this study, we initially confirmed that NAMPT expression is elevated in TNBC tissues. Subsequent experiments, including cell co-culture, exogenous cytokine treatment, and endogenous inhibition of NAMPT and its receptor expression, revealed that eNAMPT in the TNBC microenvironment likely originates primarily from TAMs. We demonstrated that eNAMPT secreted by TAMs enhances the migratory, invasive capabilities, and acquisition of stemness in TNBC cells. Conversely, inhibition of eNAMPT expression or reduction of its levels using degraders results in attenuated tumor growth and metastatic potential. Furthermore, eNAMPT has been shown to promote the differentiation of chronic lymphocytic leukemia monocytes into M2 macrophages, sustaining cancer cell survival and suppressing T cell proliferation 45. This collective evidence underscores the intricate relationship between eNAMPT and M2 macrophages in TNBC. Exogenous stimulation of cancer cells with eNAMPT in the absence of enzyme substrates has been shown to activate specific intracellular signaling pathways, such as STAT3, NF-κB, Akt, and p38, within minutes. This rapid response suggests that eNAMPT likely interacts with and activates cell-surface receptors. Manago *et al.* demonstrated that eNAMPT can induce pulmonary inflammation through direct engagement with Toll-like receptor 4 (TLR4) 46. However, TLR4 is not the sole receptor for eNAMPT. eNAMPT has also been reported to selectively inhibit human immunodeficiency virus (HIV) infection of monocytes through a direct interaction with CCR5. Phenotypic experiments conducted using HeLa cells and B16 melanoma cells with stable overexpression of CCR5 confirmed that eNAMPT binds to CCR5 and acts as a natural antagonist of this receptor 47. In non-tumoral contexts, eNAMPT has been shown to promote muscle stem cell proliferation by acting on CCR5, facilitating muscle regeneration 24. In the context of this study, we found that eNAMPT derived from TAMs binds to the receptor CCR5, inducing EMT and promoting the invasion, metastasis, and acquisition of cancer stem cell-like properties in TNBC cells. The impact of TAMs on the malignant biological behavior of TNBC cells is mediated by eNAMPT, as evidenced by the effects of CCR5 knockdown or eNAMPT inhibition. These findings suggest that eNAMPT produced by TAMs and its receptor CCR5 play pivotal roles in TNBC progression.

Our study provides evidence that eNAMPT derived from TAMs activates STAT3 signaling in a sustained manner, thereby enhancing the EMT and CSC-like characteristics of TNBC cells. This finding is in concordance with previous research documenting the role of STAT3 signaling in the crosstalk between TAMs and cancer cells within the tumor microenvironment TME. For instance, TAMs in hepatocellular carcinoma have been shown to produce IL-6, which activates the STAT3 pathway, promoting the growth of CSCs and tumor progression 48. Similarly, STAT3 phosphorylation induced by TAM-derived IL-6 has been implicated in EMT and the increased motility and invasion of CRC cells, as well as in circulating tumor cell (CTC)-mediated metastasis 39, 49. A recent study also highlighted the importance of STAT3 signaling activated by TAM-derived IL-6 in CSC enrichment and tumor growth in breast cancer 23. Our research underscores the critical role of STAT3 signaling in the aggressive behavior of tumors. While phosphorylation and dimerization of STAT3 are essential for its nuclear translocation and gene transcription, our study reveals that unphosphorylated STAT3 can also form dimers and modulate gene expression. We found that both TAMs and eNAMPT can induce acetylation of STAT3, which stabilizes the dimer and affects its DNA binding and transcriptional activity. Notably, acetylation at lysine 685 (K685) is crucial for the stable assembly of STAT3 dimers, but it does not influence the phosphorylation of STAT3 at tyrosine 705 (Y705). Prior research has indicated that nuclear CD44/acetylated-STAT3 complexes enhance the outgrowth of cells into structures resembling CSCs, promoting tumor formation 27. These cells foster EMT and metastasis, leading to a worsened prognosis. Our results suggest that post-translational modifications of STAT3, particularly acetylation, play a critical role in maintaining the stemness of cancer cells, which could make it a potential target for therapeutic intervention.

Research has established that activated STAT3, through its interaction with DNA methyltransferase 1 (DNMT1), can enhance the CpG island methylation of several tumor-suppressor genes, thereby contributing to their silencing 50. In our study, we utilized mass spectrometry to analyze the immunoprecipitated STAT3 protein complex and identified a reactivation of DIRAS2 expression. We found that reducing STAT3 acetylation can restore DIRAS2 gene expression in malignancies characterized by promoter methylation, as acetylated STAT3 directly binds to the DIRAS2 promoter. This is significant given that the majority of breast tumors exhibit downregulated DIRAS2 expression, a maternally imprinted tumor-suppressor gene that is normally expressed in the breast epithelium 51. DIRAS2, as a member of the RAS-related GTPases, is known to interact with various proteins and modulate multiple signaling pathways 52, 53. Specifically, STAT3 is a key regulator of CCL2 expression, and it has been reported to directly bind to the promoter of CCL2 to promote its transcription 54. DIRAS2 can block the NF-κB of activated B cell signaling pathway, thereby affecting the expression of cyclin and inducing G0/G1 phase arrest 52. Since the NF-κB signaling pathway is associated with the regulation of the expression of a variety of genes, including CCL2, DIRAS2 may affect CCL2 expression through this mechanism. Furthermore, DIRAS2 has been identified as a tumor suppressor gene in cutaneous melanoma by inhibiting the Wnt/β-catenin signaling pathway and is associated with immune infiltration 55. This may imply that DIRAS2 regulates tumor-related cytokine expression by affecting the Wnt/β-catenin signaling pathway. Our hypothesis is that DIRAS2 could potentially inhibit STAT3 by disrupting its dimerization, preventing its nuclear entry, or inhibiting its binding to the promoter regions of target genes. Our findings also revealed a novel feedback loop between TAMs and cancer cells. In TNBC cells co-cultured with TAMs, the ablation of DIRAS2 increased macrophage infiltration in a CCL2-dependent manner. CCL2 is well-known for its ability to attract macrophages into the tumor microenvironment. In some cases, these recruited macrophages assume a phenotype that supports tumor growth, angiogenesis, and immune evasion, contributing to a pro-tumorigenic environment 56, 57. This observation is particularly intriguing as it suggests a potential role for DIRAS2 in modulating the TME. Although few studies have explored this specific interaction, previous research has indicated that the DIRAS family can induce cellular autophagy, which may alter cellular metabolism and influence macrophage polarization 58. We hypothesize that this mechanism could be closely related to our observations and warrants further investigation. In summary, our study highlights the epigenetic impact of STAT3 acetylation on DIRAS2 expression and reveals a potential feedback loop involving TAMs and cancer cells. These findings not only expand our understanding of the molecular mechanisms underlying TNBC progression but also suggest potential therapeutic targets for modulating the TME.

### Limitations

While our study provides valuable insights into the role of the eNAMPT/Ac-STAT3/DIRAS2 axis in TNBC and its potential as a therapeutic target, there are several limitations that warrant acknowledgment. Although experiments demonstrate the effects of eNAMPT on TNBC cells, the translation of these findings to human patients requires further investigation. In future studies, it will be important to address these limitations by expanding the cell line models used, conducting *in vivo* studies, increasing sample sizes, and validating our findings in clinical trials. Exploring the interactions of eNAMPT with other components of the TME and assessing the long-term effects of targeting the eNAMPT/CCR5/STAT3/DIRAS2 axis will be crucial for advancing our understanding of TNBC biology and developing effective therapies.

## Conclusion

Our research has uncovered the presence of a significant cytokine crosstalk feedback loop between TAMs and cancer cells, which plays a crucial role in the initiation, progression, and metastasis of TNBC. The signaling cascade involving eNAMPT, CCR5, STAT3, and DIRAS2 represents a potential therapeutic target for TNBC patients. By targeting this cascade, it may be possible to disrupt the pro-tumorigenic interactions within the tumor microenvironment, offering a novel strategy for the treatment of this aggressive cancer subtype. Further research is warranted to explore the clinical applicability of these findings and to develop targeted therapies that could improve outcomes for patients with TNBC.

## Supplementary Material

Supplementary figures.

## Figures and Tables

**Table 1:** The sequences of primers for RT-qPCR.

Gene	Primer sequence (5' to 3')	
	Forward Primer	Reverse Primer
CCL2	ATCAATGTGACGGCAGGGAAA	TCGAAACCTCTCTGCTCTAACAC
CD163	AGGAGAGAACTTAGTCCACCA	ATGGCCTCCTTTTCCATTCCA
NAMPT	GGCACCACTAATCATCAGACCTG	AAGGTGGCAGCAACTTGTAGCC
CCR5	GTCTACTTTCTCTTCTGGACTCC	CCAAGAGTCTCTGTTGCCTGCA
DIRAS2	AAAGGCACATTCCGGGAGAG	GTGCCCTTTGGAGATGGACA
E-cadherin	TGGAACAGGGACACTTCTGC	CCCCGTGTGTTAGTTCTGCT
GAPDH	ACCACAGTCCATGCCATCAC	TCCACCACCCTGTTGCTGTA
HLA-DR	TCACGTGGCTTCGAAATGGA	TCCACCCTGCATCGTAAAC
IL-1β	CAGCAGTTGGTCATCTCTTGG	GCTTAGAACCTCGCCTCCTT
IL-6	TCAATATTAGAGTCTCAACCCCCA	GAGAAGGCAACTGGACCGAA
IL-10	AAGCCTTGTCTGAGTGAT	CATTCTTCACCTGCTCCA
iNOS	CCCTTCAATGGTTGGTACATGG	ACATTGATCTCCGTGACAGCC
Nanog	GTCTCCACACATCAGCACA	TTCGCCTCTTGACATTCTCCT
N-cadherin	TGCGGTACAGTGTAACTGGG	GAAACCGGGCTATCTCTGCTGG
TGF-β	GACTACTACGCCAAGGAG	TGAGGTATCGCCAGGAAT
TNF-α	TCTTCTCCTTCCTGATCGT	GCTACAGGCTTGTCACTC
Snail	GACGCCATCAACACCGAGTT	CTTTGTCGTTGGTTAGCTGGT
SOX2	TGAGCGCCCTGCAGTACAA	GCTGCGTAGGACATGCTGTAG
4-Oct	CCCCTGGTGCCGTGAAG	GCAAAGCTCGAGTTCTTTCTG

**Table 2:** Correlation between CD163 expression and clinicopathological parameters (*n*=48).

Characteristics	n (%)	CD163 expression		
		low	high	*P*
Age (years)				**0.039**
< 50	14 (29.1)	4	10	
≥ 50	34 (70.9)	11	23	
TNM stage				**0.002**
Ⅰ	15 (31.2)	9	6	
Ⅱ	20 (41.7)	8	12	
Ⅲ-Ⅳ	13 (27.1)	1	12	
Ki-67 (%)				0.473
< 14	1 (2.1)	1	0	
≥ 14	47 (97.9)	17	30	
