# Supplementary material for: eNAMPT/Ac-STAT3/DIRAS2 Axis Promotes Development and Cancer Stemness in Triple-Negative Breast Cancer by Enhancing Cytokine Crosstalk Between Tumor-Associated Macrophages and Cancer Cells
Source: Int J Biol Sci. 2025 Feb 18;21(5):2027–47. doi: 10.7150/ijbs.103723 (PMC11900821; doi:10.7150/ijbs.103723)

Supplementary figures

Fig. S1 THP-1-induced M2 macrophages promote TNBC cell migration and invasion. (A) Western blot analysis confirms the expression of Arginase 1 in THP-1 cells and THP-1-induced M2 macrophages, indicating the M2 polarization status. (B-C) Wound healing and Transwell invasion assays (magnification, 100×, scale bar=100 μm) were conducted to evaluate the impact of THP-1-induced M2 macrophages on MDA-MB-231 cell migration and invasion. **P* < 0.05.

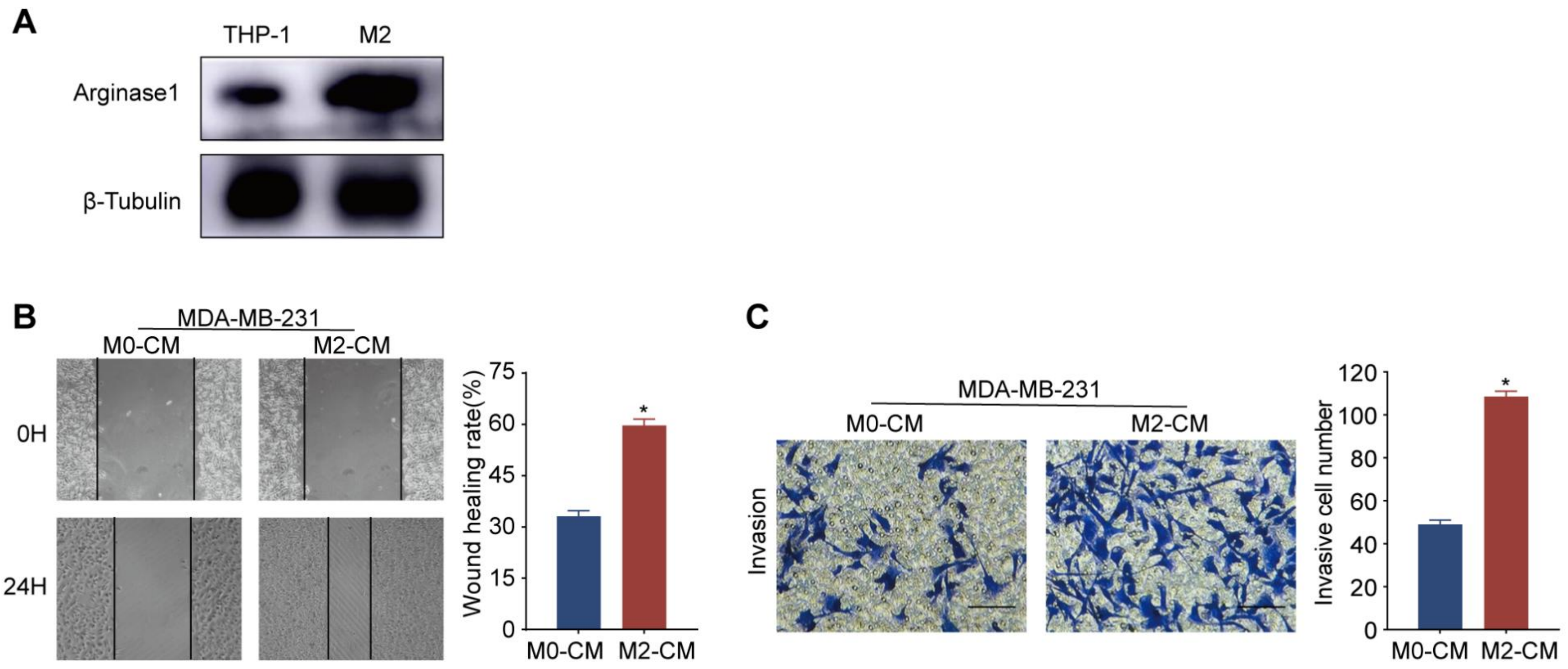

Fig. S2 Impact of M2 macrophages on TNBC cell migration and invasion and expression analysis of NAMPT in breast cancer. (A) Venn diagram displaying the intersection of differentially expressed genes in two TNBC cell lines following co-culture with macrophages. (B-C) Correlation analysis reveals a positive association between NAMPT expression and macrophage infiltration within the TNBC microenvironment. (D) NAMPT expression exhibits a positive correlation with the M2 macrophage markers CD163 and CD206 in TNBC tissues. (E) Comparative expression analysis of NAMPT across normal tissues and various breast cancer subtypes. (F) Immunohistochemical staining depicts NAMPT protein levels in normal and breast cancer tissues, with staining intensity indicated. (G) NAMPT expression is profiled across different breast cancer cell line subtypes, including TNBC. (H) Wound-healing and Transwell assays (magnification, 100×, scale bar=100 μm) assess the migration and invasion of SUM159 cells under different conditions: cultured alone, co-cultured with M2 macrophages, or co-cultured with M2 macrophages where NAMPT expression is silenced. *$P < 0.05$, **$P < 0.01$.

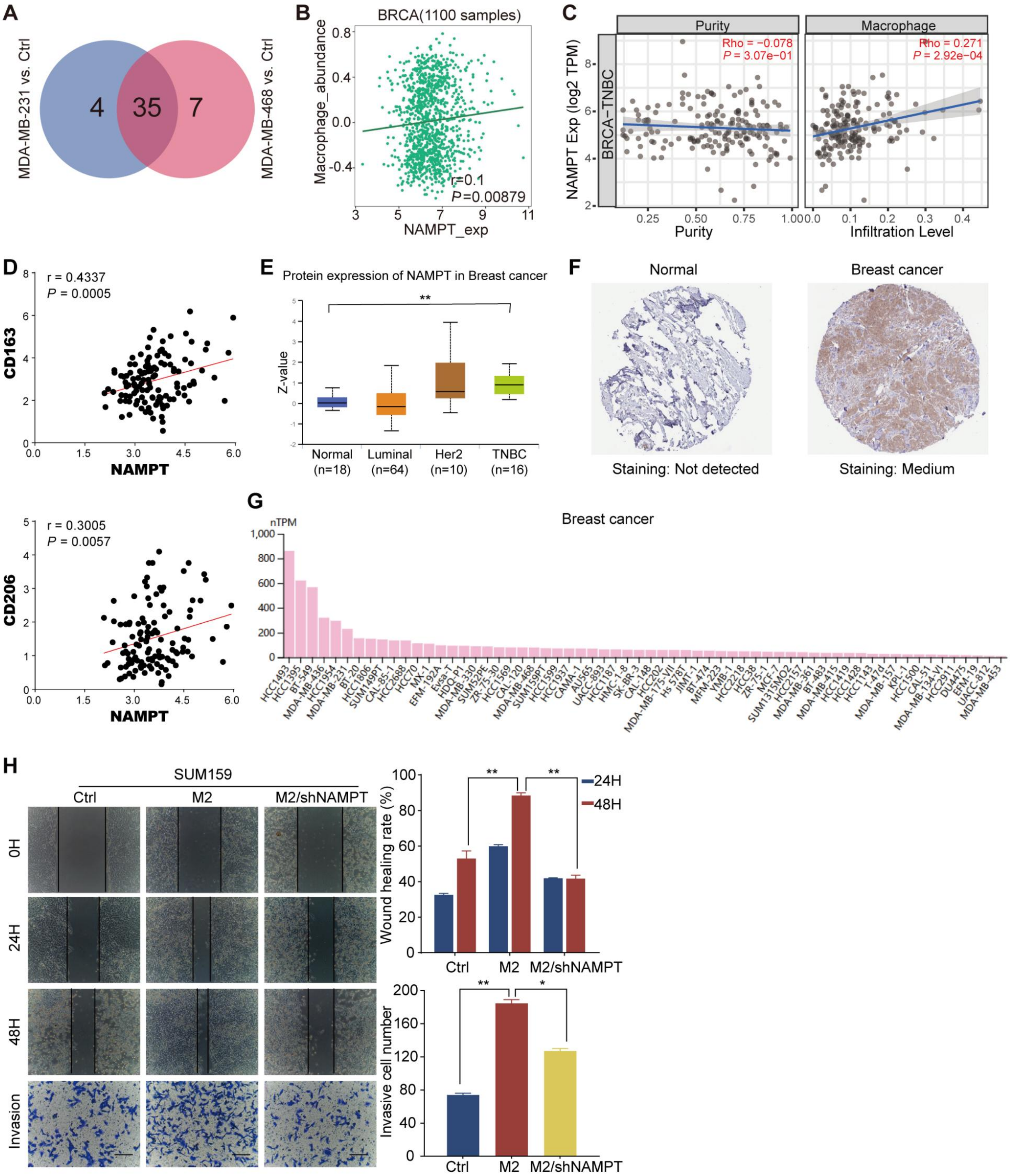

Fig. S3 Effect of eNAMPT or shCCR5 on migration, invasion, and stemness in MDA-MB-231 and SUM159 cells. (A) Analysis demonstrates a positive correlation between CCR5 expression and macrophage infiltration within the breast cancer microenvironment. (B) Comparative assessment of CCR5 expression in breast cancer tissues versus normal tissues. (C) Correlation analysis indicates a positive association between CCR5 and NAMPT expression in TNBC tissues. (D-E) Wound-healing and Transwell assays (magnification, 100×, scale bar=100 μm) were used to evaluate the effects of eNAMPT or shCCR5 on the migration and invasion of MDA-MB-231 and SUM159 cells. (F) A microsphere formation assay (magnification, 100×, scale bar=100 μm) was conducted to determine the stemness of TNBC cells (MDA-MB-231 and SUM159) following eNAMPT treatment or CCR5 knockdown. *$P < 0.05$, **$P < 0.01$, ***$P < 0.001$.

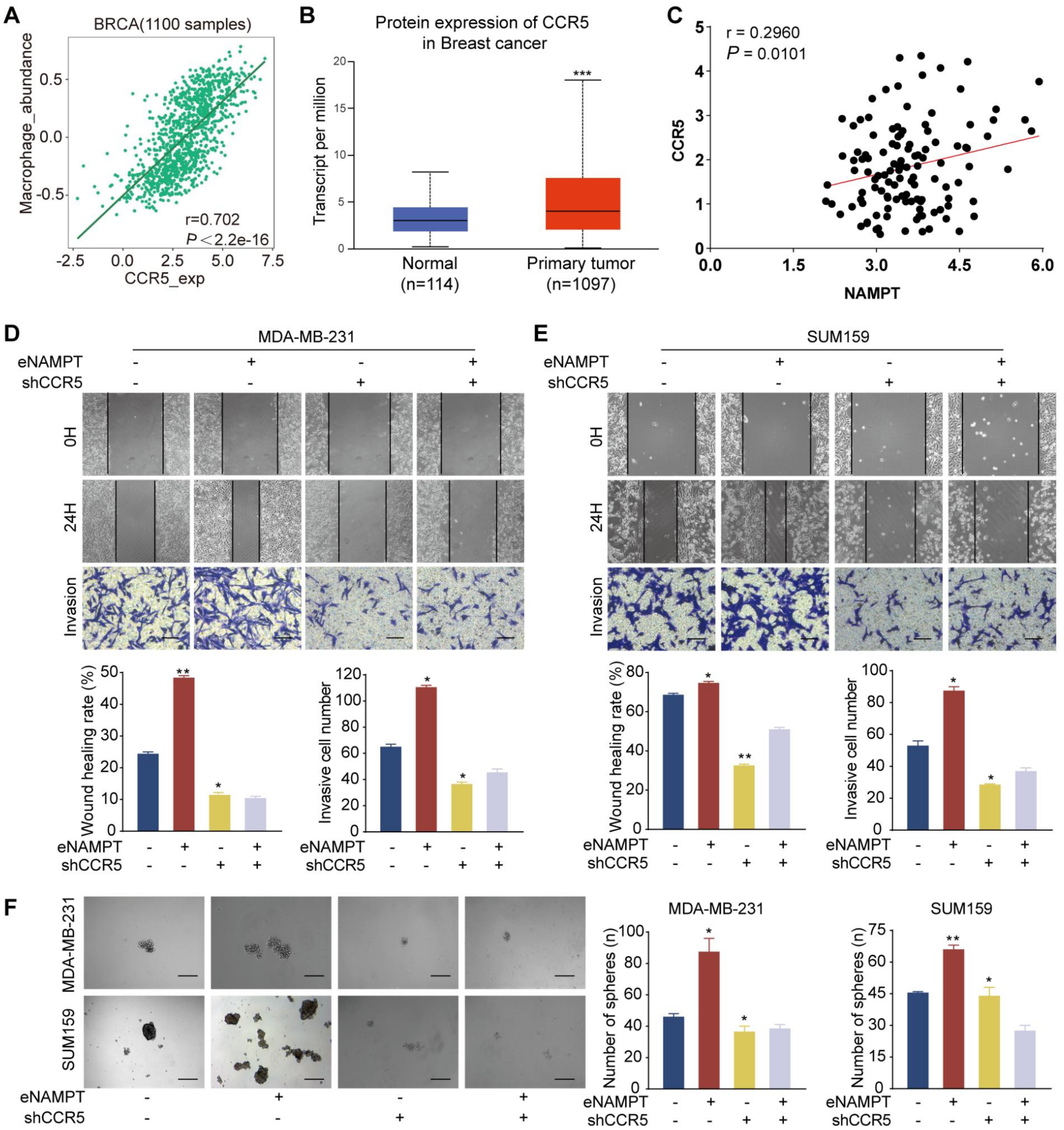

Fig. S4 Impact of M2 macrophages and STAT3 inhibition on TNBC cell migration, invasion, and stemness. (A) Wound-healing and Transwell assays (magnification, 100×, scale bar=100 μm) were conducted to assess the migration and invasion of TNBC cells (MDA-MB-231 and SUM159) cultured alone or co-cultured with M2 macrophages, with or without the addition of Stattic, a STAT3 inhibitor. (B) A microsphere formation assay (magnification, 100×, scale bar=100 μm) was used to evaluate the stemness of TNBC cells (MDA-MB-231 and SUM159). *$P < 0.05$, **$P < 0.01$, ***$P < 0.001$.

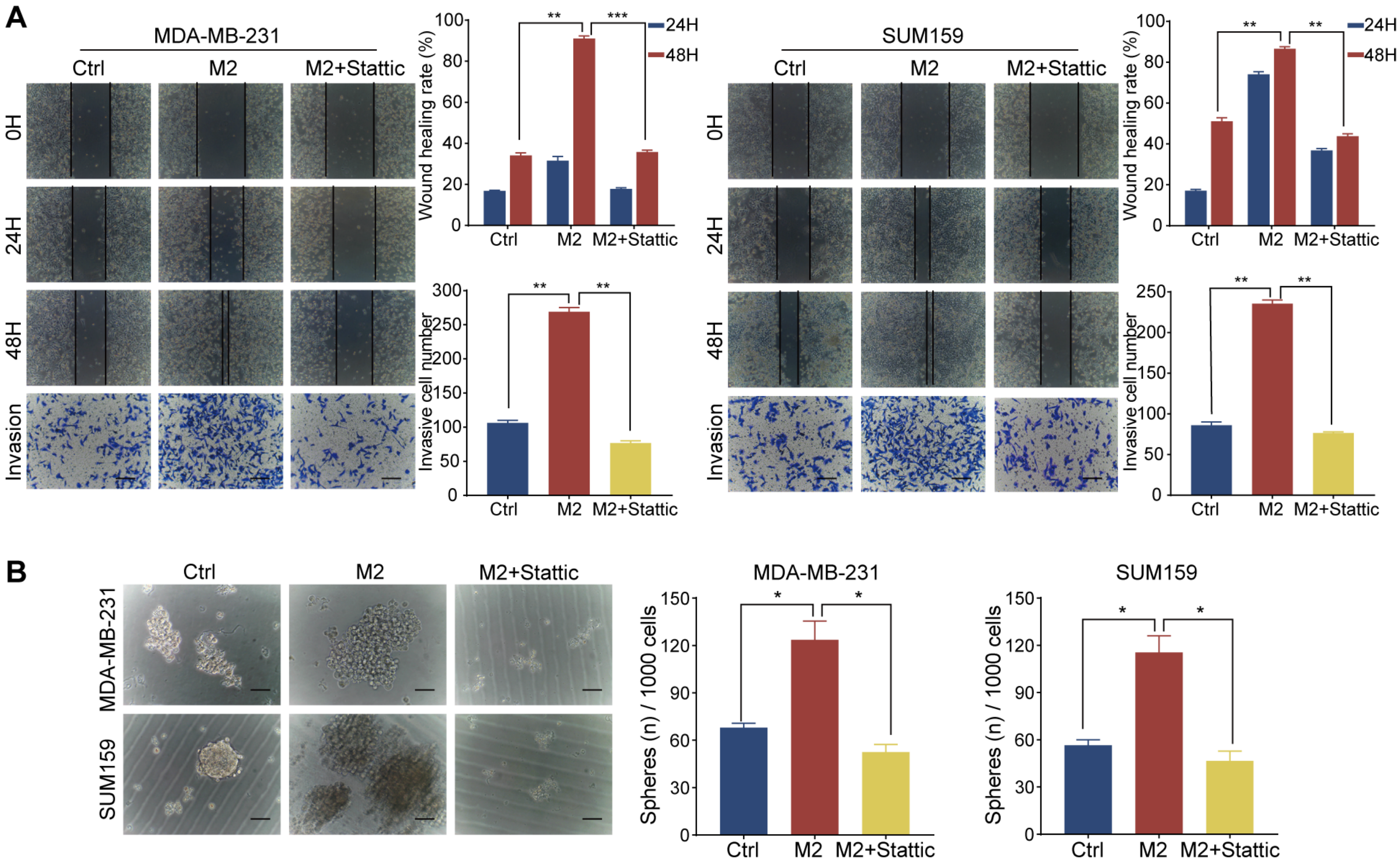

Supplement: Supplementary file 1 — Supplementary figures. [file ijbsv21p2027s1.pdf]
